# Conformational eyelid disorders in dogs under primary veterinary care in the UK - Epidemiology and clinical management

**DOI:** 10.1371/journal.pone.0326526

**Published:** 2025-06-30

**Authors:** Dan G. O’Neill, Minna P. Mustikka, Dave C. Brodbelt, David B. Church, Vilma Vaattovaara

**Affiliations:** 1 Pathobiology and Population Sciences, The Royal Veterinary College, Hatfield, Herts, United Kingdom; 2 Department of Equine and Small Animal Medicine, Faculty of Veterinary Medicine, University of Helsinki, Finland; 3 Clinical Sciences and Services, The Royal Veterinary College, Hatfield, Herts, United Kingdom; Wrocław University of Environmental and Life Sciences: Uniwersytet Przyrodniczy we Wroclawiu, POLAND

## Abstract

**Introduction:**

Natural eyelid conformation is essential for normal function and health of the ocular surface. However, many modern dog types are deliberately bred for abnormal eyelid conformation associated with severe health and welfare issues. This study aimed to report the prevalence, demographic risk factors and clinical management under primary veterinary care for conformational eyelid disorders in dogs.

**Methods:**

The study explored the anonymised clinical records of all dogs under UK primary veterinary care within the VetCompass Programme during 2019. Risk factor analysis used multivariable logistic regression modelling.

**Results:**

From 2,250,417 dogs under primary veterinary care in 2019, the analysis included a random sample of 3,029 confirmed conformational eyelid disorder cases that included 2,752 (90.86%) entropion and 344 (11.36%) ectropion cases. After accounting for the subsampling process, the annual prevalence for overall conformational eyelid disorder in dogs was 0.36% (95% CI: 0.35–0.37). The annual prevalence for entropion in dogs overall was 0.33% (95% CI: 0.32–0.34). Breeds with highest annual prevalence for entropion were Shar-Pei (15.41%, 95% CI 14.00–16.91), Chow Chow (9.28%, 95% CI 7.64–11.14) and Neapolitan Mastiff (6.88%, 95% CI 3.02–13.14). The estimated annual prevalence for ectropion in dogs overall was 0.04% (95% CI: 0.04–0.05). Breeds with the highest annual prevalence for ectropion were Neapolitan Mastiff (4.30%, 95% CI 1.41–9.77), Saint Bernard (1.72%, 95% CI 0.86–3.05) and Basset Hound (1.59%, 95% CI 0.94–2.49). Surgical management was carried out for 414/2275 (18.20%) of the incident (2019) entropion cases and 12/305 (3.93%) of the incident (2019) ectropion cases.

**Conclusions:**

Normalisation of conformation-related health issues in certain high profile dog breeds have been discussed as a canine welfare priority for over half a century. The current results suggest that substantial work remains to be done to ensure good innate health for all dogs.

## Introduction

Natural eyelid conformation is essential for good function and health of the ocular surface [1]. The most important conformational eyelid disorders are entropion, i.e., inversion of the eyelid margin, and ectropion, i.e., eversion of the eyelid margin [1,2]. Entropion may result from uneven tension between the orbicularis oculi muscle and the muscles opening the eyelids. The severity of entropion may be influenced by several factors including the length of the palpebral fissure, conformation of the skull, the orbital anatomy, sex, and the extensiveness of folds of facial skin around the eyes. Similarly, the orbital anatomy, length of the eyelids and excessive facial skin folds, heavy ears and an unstable lateral canthus can result in ectropion and lateral canthal entropion [2–4]. While the orbicularis oculi muscle encircling the palpebral fissure acts to shut the eyelids, the elliptical palpebral fissure closes in a horizontal slit due to the ligamentous attachments of the eyelids to the medial and lateral orbital wall via the medial canthal ligament, lateral canthal ligament and *retractor anguli oculi lateralis* [1,2]. Relative conformational abnormalities, such as weakness/laxity, excessive tightness and/or misdirected attachment in these structures has been reported to potentially result in medial and/or lateral canthal entropion [1,2,5,6]. Entropion often accompanies ectropion in macroblepharon when the lateral portion of overly long and sagging ectropic eyelid margins invert [1,2]. Entropion and/or ectropion are presumed to be inherited polygenically in several dog breeds [1–3,7]. Both entropion and ectropion are considered as disorders that threaten the welfare and retention of eyesight of affected individuals due to the discomfort and inflammation associated with hair contact on the densely innervated ocular surface and also from painful corneal ulceration ranging from superficial ulcers to perforation of the globe with the risk of loss of vision or loss of the globe [1,2,6,8–11]

Much of the information published to date on conformational eyelid disorders in dogs concentrated on describing various surgical approaches and their success rates in diminishing negative effects from these disorders. While such studies are important to share information on how to address existing problems at the level of the afflicted individual, most papers describe referral-based caseloads of already affected animals and fail to offer population-level guidance on how to promote better dog acquisition decisions away from choosing dog types with extreme eyelid conformation [9,11–13]. Substantial information regarding presumed hereditary eye diseases is recorded during eye examinations performed for breeding purposes [14,15]. Nationally recorded data are collected by the European College of Veterinary Ophthalmologist (ECVO) and the American College of Veterinary Ophthalmologists (ACVO), but only information from ACVO is shared publicly [7]. This ACVO ‘Blue Book’ source has an intrinsic US geographic bias but also lacks information from companion dogs in the wider population that are not undergoing eye examinations for breeding purposes or are not diagnosed with conformational eyelid issues severe enough to warrant referral for specialist care. Consequently, there is an important data gap on the occurrence of conformational eyelid disorders in the general population of dogs outside of specific breeding programmes.

Secondary application of anonymised primary care veterinary clinical records as a research resource is now widely accepted as a key contributor to the overall evidence base for companion animal health and welfare [16]. However, there remains limited published information from caseloads under primary veterinary care on prevalence and breed-based risk factors for conformational eyelid disorders in dogs overall, although primary care studies have been published on other ophthalmic disorders including keratoconjunctivitis sicca, ulcerative keratitis and prolapsed nictitating membrane gland (PNMG) [17–19]. In addition, several disorders affecting the head that are linked with breed-related conformations in dogs, such as excessive skin folds and brachycephalism, have also been reported using primary care veterinary data and have contributed evidence to support growing international concerns about the welfare of dogs with extreme conformations [20–26]. Dramatically rising demand for many breeds defined by their extreme conformation, especially brachycephaly [27], led to the establishment of an international body in 2019, the International Collaborative on Extreme Conformations in Dogs (ICECDogs). The stated mission of ICECDogs is to work together to minimise welfare issues resulting from extreme conformations in dogs by seeking out and applying evidence-based canine and human approaches [26]. ICECDogs defines extreme conformation in dogs as “a physical appearance that has been so significantly altered by humankind away from the ancestral natural canine appearance that affected dogs commonly suffer from poor health and welfare, with negative impacts on their quality and quantity of life” [26]. Conformation related eyelid disorders meet this definition as an extreme conformation but deficiencies in their quantity and quality of the available evidence in the general population of dogs is widely acknowledged as contributing to the relatively slow progress to date towards eradicating this extreme conformation from domestic dogs [28].

There is evidence of predisposition to conformational eyelid disorders in several dog breeds. A questionnaire study of owners of pedigree dogs registered with the UK Kennel Club reported an overall prevalence of 0.61% for entropion [29]. Based on clinical information from primary care veterinary data in the UK, entropion was the most common disorder diagnosed in Shar Pei dogs with a prevalence of 17.88%, although ectropion did not feature among the 30 most common disorders in that breed [22]. Among English Bulldogs in the UK, entropion was the 13^th^ most common disorder with a prevalence of 3.6%, although ectropion was not among the 29 most common disorders that were reported in that paper [18]. Despite these high breed-specific prevalence values, neither entropion nor ectropion featured among the 70 most diagnosed disorders in dogs overall in the UK, suggesting that some breeds carry extremely high predisposition for these conformational eyelid disorders.

The current study aimed to report the prevalence and demographic risk factors for overall conformation related disorders and also separately for entropion and ectropion in dogs using anonymised veterinary clinical data from the VetCompass Programme in the UK [30]. Particular focus was placed on breed associations with disease. The study also aimed to report on clinical management approaches commonly undertaken in the veterinary primary care setting. These results could assist veterinary practitioners, welfare scientists, breeders and owners, and also contribute to task forces and ethics groups working on regulations and legislation regarding dog breeding, by providing a stronger evidence base to understand, predict, prevent and better manage conformational eyelid disorders in dogs [31].

## Methods

The study population included all dogs under primary veterinary care at clinics participating in the VetCompass Programme during 2019. Dogs under veterinary care were defined as those with ≥ 1 electronic health record (EHR) (free-text clinical note, treatment or bodyweight) recorded during 2019. VetCompass collates de-identified EHR data from approximately 25% of UK primary-care veterinary practices for epidemiological research [30]. These practices are mainly constituent within large veterinary groups in the UK and therefore may not fully represent all veterinary practices in the UK. Data fields for each animal include fixed values for species, breed, date of birth, sex and neuter status along with date-specific information on free-form text clinical notes, bodyweight and treatment.

A cohort study design with a cross-sectional analysis was used to estimate the one-year (2019) prevalence of conformational eyelid disorder (entropion and ectropion) and to explore associations with demographic risk factors in this population. Based on prior evidence for 0.61% prevalence of entropion in dogs in the UK [29], power calculation estimated that a study sample of 23,241 dogs was needed to estimate prevalence with 0.1% acceptable margin of error at a 95% confidence level from a national UK population of 11 million dogs [32,33]. Ethics approval was obtained from the RVC Ethics and Welfare Committee (reference SR2018−1652). All methods were performed in accordance with the relevant guidelines and regulations. The study is reported in accordance with ARRIVE guidelines [34].

The case definition for a conformational eyelid disorder required evidence in the clinical records for a final diagnosis of entropion and/or ectropion (or synonym) at any date from Jan 1, 2019 to Dec 31, 2019. Case-finding involved initial screening of all 2,250,417 study dogs for candidate conformational eyelid disorder cases. Search terms used to screen the clinical free-text and treatment free-text fields were entrop*, entropion~2, hotz*, holtz*, hots-cel*, “diamond eye”, arrowhead, etrop*, enrtop*, intropi*, antropi*, “inversion of eyelid”, “conformational eyelid”, microblep*, ectrop*, ectropion~2, “diamond eye”, extrop*, eurybleph*, macrob*, etrop*, “eversion of eyelid” and ecrtopi*. The clinical notes of a random sample of candidate animals were manually reviewed to evaluate for case inclusion. Additional information extracted for confirmed conformational eyelid disorder cases included the date of diagnosis, clinical signs on presentation regardless of whether it was stated that these were related to the conformational eyelid disorder, and diagnostic approaches and treatments. All dogs that were not screened as candidate cases were included as non-cases in the risk factor analysis.

Breed descriptive information entered by the participating practices was cleaned and mapped to a VetCompass breed list derived and extended from the VeNom Coding breed list that included both recognised purebred breeds and also designer crossbreed breed terms [35]. A *breed purity* variable categorised all dogs of recognisable breeds as ‘purebred’, dogs with contrived names generated from two or more purebred breed terms as ‘designer crossbreed’ crossbreds (purposely bred crossbreeds) and dogs recorded as mixes of breeds but without a contrived name as ‘general crossbred’ [36]. A *breed* variable included individual pure breeds and designer hybrids represented by over 10,000 dogs in the overall study population or with ≥ 10 conformational eyelid disorder cases, along with a grouping of all remaining breeds and also general crossbred dogs. This approach was taken to facilitate statistical power for the individual breed analyses [37].

Breeds were characterised by ear carriage based on pinnal phenotypes typically described for each breed [36,38,39]. The categories of ear carriage included erect (also known as prick or upright, e.g., German Shepherd Dog), semi-erect (also known as cocked or semi-pricked, e.g., Rough Collie), v-shaped drop (also known as folded, e.g., Hungarian Vizsla), pendulous (also known as drop or pendant, e.g., Basset Hound) and unspecified [40]. Breeds were also characterised by coat length (short, medium, long, uncategorised), skull shape (dolichocephalic, mesocephalic, brachycephalic, uncategorised), brachycephalic severity (mild, moderate and severe) and spaniel type (spaniel type purebred, non-spaniel type purebred, spaniel type designer crossbred, non-spaniel designer crossbred, general crossbred) [27]. A Kennel Club recognised breed variable categorised breeds as recognised by the Kennel Club or not. A *Kennel Club breed group* variable classified breeds recognised by the UK Kennel Club into their relevant breed groups (Gundog, Hound, Pastoral, Terrier, Toy, Utility and Working) and all remaining types were classified as non-Kennel Club recognised [36].

A sex-neuter variable described the recorded status at the final available EHR value (female entire, female neutered, male entire, male neutered, unrecorded). Adult bodyweight was defined as the median of all bodyweight (kg) values recorded for each dog after reaching 18 months old and was categorised as: < 10.0, 10.0 to < 15.0, 15.0 to < 20.0, 20.0 to < 25.0, 25.0 to < 30.0, 30.0 to < 40.0, 40.0 to < 50.0, 50.0 to < 60.0 and ≥ 60.0. Age (years) was defined for each dog on December 31, 2019 and was categorised in one year bands to 24 years.

Following internal validity checking and data cleaning in Excel (Microsoft Office Excel 2013, Microsoft Corp.), analyses were conducted using Stata Version 16 (Stata Corporation). Annual prevalence with 95% confidence interval (CI) described the probability of diagnosis at any point during 2019. Because the sampling design involved verification of a subset of the overall candidate cases, the prevalence and 95% CI were based on the number of confirmed cases from the sampling proportion of the overall study population both overall and for each breed. The CI estimates were derived from standard errors, based on approximation to the binomial distribution [41]. Results for clinical management were reported descriptively. Risk factor analysis used binary logistic regression modelling to evaluate univariable associations between risk factors (*breed, breed purity, Kennel Club recognised breed, Kennel Club breed group, skull shape, brachycephalic severity, spaniel type, coat length, ear carriage, adult bodyweight, age, sex-neuter, veterinary group*) and conformational eyelid disorder overall (analyses were repeated using entropion and ectropion separately as outcomes) during 2019. Because breed was a factor of primary interest for the study, variables that derived from the breed information and therefore were highly correlated with breed (*breed purity, Kennel Club recognised breed, Kennel Club breed group, skull shape, brachycephalic severity, spaniel type, coat length, ear carriage, adult bodyweight*) were not considered in the initial breed multivariable modelling. Instead, each of these derived variables individually replaced the *breed* variable in the main breed-focused model to evaluate their effects after taking account of the other variables. Risk factors with liberal associations in univariable modelling (*P *< 0.2) were taken forward for multivariable evaluation. Model development used manual backwards stepwise elimination. Pair-wise interaction effects were evaluated for the final model variables [42]. The area under the ROC curve and McKelvey & Zavoina Pseudo-R² were used to evaluate the quality of the model fit [42,43]. Statistical significance was set at *P *< 0.05.

## Results

### Descriptive

#### Conformational eyelid disorders overall.

Text searches of an overall study population of 2,250,417 dogs under primary veterinary care in 2019 yielded 13,423 candidate cases of conformational eyelid disorder with some evidence of entropion and/or ectropion in 2019. Manual checking of a random sample of 5,000/13,423 (37.25% sampling proportion) candidate cases identified 3,029 confirmed conformational eyelid disorder cases during 2019 from a notional 838,269 study sample (i.e., 37.25% sampling proportion of study population). After accounting for the subsampling protocol, the estimated annual prevalence for conformational eyelid disorder in dogs overall was 0.36% (95% CI: 0.35–0.37).

**Fig 1:**
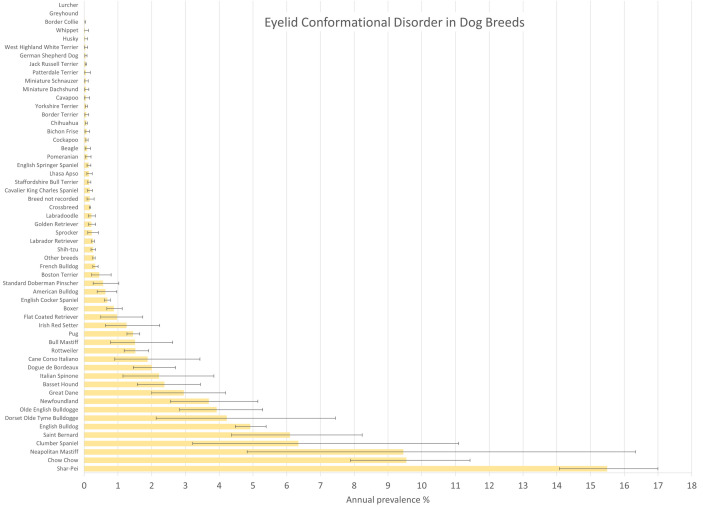
Annual prevalence (%) of conformational eyelid disorder in dog breeds under primary veterinary care in the VetCompass Programme in the UK in 2019. The horizontal bars represent 95% confidence intervals.

Breeds with the highest annual prevalence for conformational eyelid disorder overall were Shar-Pei (15.49%, 95% CI 14.08–17.00), Chow Chow (9.55%, 95% CI 7.88–11.43), Neapolitan Mastiff (9.45%, 95% CI 4.83–16.33), Clumber Spaniel (6.34%, 3.22–11.09), Saint Bernard (6.10%, 95% CI 4.37–8.24) and English Bulldog (4.92%, 95% CI 4.48–5.39) (Fig 1). Among the 3,029 conformational eyelid disorder cases, the most common breeds were English Bulldog (n = 433, 14.30%), Shar-Pei (377, 12.45%), Crossbreed (350, 11.55%), English Cocker Spaniel (244, 8.06%) and Pug (229, 7.56%).

From conformational eyelid disorder cases with data available for that variable, 2,606 (86.35%) were purebred, 1,401 (46.62%) were female and 914 (30.42%) were neutered. Conformational eyelid disorder cases had a median adult bodyweight of 23.60 kg (IQR: 13.30–30.65, range 2.50–95.00) and median age was 2.95 years (IQR: 1.43–6.29, range 0.04–17.41). (Table 1). Of the dogs that were not conformational eyelid disorder cases with data available on the variable, 1,540,146 (69.26%) were purebred and 1,061,592 (47.87%) were female, 971,553 (43.81%) were neutered. The median adult bodyweight for conformational eyelid disorder cases was 13.65 kg (IQR: 8.40–24.40, range 0.38–106.00) and the median age was 5.25 years (IQR: 2.26–9.00, range 0.00–24.98). (Table 1).

**Table 1:** Descriptive and breed-focused multivariable logistic regression results for risk factors associated with *conformational eyelid disorder* cases overall during 2019 in dogs under primary veterinary care in the VetCompass™ Programme in the UK.

Variable	Case No. (%)	Non-case No. (%)	Odds Ratio	95% CI	Category *P*-value	Variable *P*-value
Breed						**< 0.001**
Crossbreed	350 (11.55)	534,753 (23.9)	Reference category			
Shar-Pei	377 (12.45)	5,244 (0.23)	107.38	92.51–124.64	**< 0.001**	
Chow Chow	106 (3.50)	2,373 (0.11)	60.05	48.06–75.01	**< 0.001**	
Neapolitan Mastiff	11 (0.36)	263 (0.01)	58.70	31.77–108.46	**< 0.001**	
Saint Bernard	39 (1.29)	1,356 (0.06)	38.85	27.75–54.37	**< 0.001**	
Clumber Spaniel	11 (0.36)	501 (0.02)	33.43	18.20–61.40	**< 0.001**	
English Bulldog	433 (14.30)	21,427 (0.96)	26.87	23.28–31.02	**< 0.001**	
Newfoundland	33 (1.09)	2,067 (0.09)	23.26	16.23–33.35	**< 0.001**	
Dorset Olde Tyme Bulldogge	11 (0.36)	654 (0.03)	22.51	12.28–41.28	**< 0.001**	
Italian Spinone	12 (0.40)	930 (0.04)	19.32	10.82–34.50	**< 0.001**	
Olde English Bulldogge	41 (1.35)	2,926 (0.13)	18.25	13.15–25.33	**< 0.001**	
Great Dane	30 (0.99)	2,525 (0.11)	16.90	11.61–24.6	**< 0.001**	
Basset Hound	27 (0.89)	3,827 (0.17)	11.97	8.08–17.74	**< 0.001**	
Dogue de Bordeaux	42 (1.39)	5,003 (0.22)	11.76	8.52–16.23	**< 0.001**	
Bull Mastiff	12 (0.40)	1,768 (0.08)	10.67	5.98–19.02	**< 0.001**	
Irish Red Setter	11 (0.36)	2,121 (0.09)	8.21	4.50–15.00	**< 0.001**	
Pug	229 (7.56)	39,659 (1.77)	8.05	6.81–9.53	**< 0.001**	
Rottweiler	71 (2.34)	12,938 (0.58)	8.05	6.23–10.40	**< 0.001**	
Cane Corso Italiano	10 (0.33)	1,788 (0.08)	7.02	3.73–13.20	**< 0.001**	
Flat Coated Retriever	11 (0.36)	2,640 (0.12)	6.24	3.42–11.39	**< 0.001**	
Boxer	61 (2.01)	17,316 (0.77)	5.57	4.24–7.31	**< 0.001**	
English Cocker Spaniel	244 (8.06)	95,761 (4.28)	3.82	3.24–4.50	**< 0.001**	
American Bulldog	20 (0.66)	8,541 (0.38)	3.16	2.01–4.96	**< 0.001**	
Boston Terrier	11 (0.36)	5,426 (0.24)	2.74	1.50–5.00	**0.001**	
Standard Doberman Pinscher	10 (0.33)	5,691 (0.25)	2.50	1.33–4.70	**0.004**	
Sprocker	10 (0.33)	9,301 (0.42)	1.57	0.84–2.94	0.162	
Other breeds	238 (7.86)	229,252 (10.25)	1.56	1.32–1.84	**< 0.001**	
Shih-tzu	65 (2.15)	67,018 (3.00)	1.51	1.16–1.97	**0.002**	
Labrador Retriever	141 (4.66)	153,501 (6.86)	1.44	1.18–1.75	**< 0.001**	
French Bulldog	75 (2.48)	66,531 (2.97)	1.40	1.09–1.80	**0.009**	
Breed not recorded	11 (0.36)	13,252 (0.59)	1.29	0.66–2.51	0.462	
Labradoodle	19 (0.63)	21,697 (0.97)	1.21	0.75–1.94	0.434	
Cavalier King Charles Spaniel	23 (0.76)	35,139 (1.57)	1.07	0.70–1.63	0.753	
Golden Retriever	19 (0.63)	27,331 (1.22)	0.99	0.62–1.60	0.981	
Lhasa Apso	14 (0.46)	24,462 (1.09)	0.96	0.56–1.63	0.874	
Staffordshire Bull Terrier	43 (1.42)	93,613 (4.18)	0.77	0.56–1.06	0.110	
English Springer Spaniel	25 (0.83)	51,647 (2.31)	0.75	0.49–1.13	0.170	
Pomeranian	7 (0.23)	14,798 (0.66)	0.67	0.32–1.41	0.290	
Beagle	6 (0.20)	20,167 (0.9)	0.44	0.20–10	**0.049**	
Yorkshire Terrier	14 (0.46)	53,175 (2.38)	0.44	0.26–0.76	**0.003**	
Border Terrier	6 (0.20)	24,656 (1.10)	0.41	0.18–0.91	**0.029**	
Bichon Frise	6 (0.20)	25,113 (1.12)	0.40	0.18–0.89	**0.025**	
Chihuahua	22 (0.73)	80,493 (3.60)	0.40	0.26–0.61	**< 0.001**	
Miniature Schnauzer	5 (0.17)	21,115 (0.94)	0.36	0.15–0.87	**0.023**	
Cockapoo	21 (0.69)	72,822 (3.26)	0.35	0.23–0.56	**< 0.001**	
Jack Russell Terrier	20 (0.66)	101,182 (4.52)	0.35	0.22–0.55	**< 0.001**	
Patterdale Terrier	2 (0.07)	10,739 (0.48)	0.30	0.08–1.21	0.092	
Cavapoo	3 (0.10)	14,112 (0.63)	0.29	0.09–0.89	**0.031**	
German Shepherd Dog	8 (0.26)	47,347 (2.12)	0.25	0.12–0.5	**< 0.001**	
Miniature Dachshund	4 (0.13)	24,780 (1.11)	0.22	0.08–0.59	**0.003**	
West Highland White Terrier	4 (0.13)	35,768 (1.60)	0.21	0.08–0.55	**0.002**	
Husky	2 (0.07)	17,311 (0.77)	0.18	0.04–0.70	**0.014**	
Whippet	1 (0.03)	12,750 (0.57)	0.12	0.02–0.83	**0.032**	
Border Collie	2 (0.07)	61,751 (2.76)	0.05	0.01–0.21	**< 0.001**	
Greyhound	0 (0.00)	12,630 (0.56)	N/A			
Lurcher	0 (0.00)	16,043 (0.72)	N/A			
Age (years)						**< 0.001**
<1.0	396 (13.12)	230,875 (10.40)	0.68	0.6–0.77	**< 0.001**	
1.0- < 2.0	690 (22.86)	264,614 (11.92)	Reference category			
2.0- < 3.0	441 (14.61)	212,863 (9.59)	0.84	0.75–0.95	**0.006**	
3.0- < 4.0	263 (8.71)	183,137 (8.25)	0.60	0.52–0.69	**< 0.001**	
4.0- < 5.0	242 (8.02)	177,514 (8.00)	0.60	0.52–0.69	**< 0.001**	
5.0- < 6.0	171 (5.67)	163,450 (7.36)	0.47	0.40–0.56	**< 0.001**	
6.0- < 7.0	175 (5.80)	154,492 (6.96)	0.53	0.45–0.63	**< 0.001**	
7.0- < 8.0	149 (4.94)	144,150 (6.49)	0.50	0.42–0.60	**< 0.001**	
8.0- < 9.0	126 (4.17)	134,666 (6.07)	0.47	0.39–0.57	**< 0.001**	
9.0- < 10.0	99 (3.28)	121,302 (5.47)	0.42	0.34–0.52	**< 0.001**	
10.0- < 11.0	94 (3.11)	108,221 (4.88)	0.48	0.39–0.60	**< 0.001**	
11.0- < 12.0	55 (1.82)	93,193 (4.20)	0.34	0.26–0.45	**< 0.001**	
12.0- < 13.0	46 (1.52)	78,981 (3.56)	0.36	0.27–0.49	**< 0.001**	
13.0- < 14.0	28 (0.93)	60,798 (2.74)	0.31	0.21–0.46	**< 0.001**	
14.0- < 15.0	25 (0.83)	42,628 (1.92)	0.44	0.29–0.66	**< 0.001**	
15.0- < 16.0	16 (0.53)	25,896 (1.17)	0.51	0.31–0.84	**0.008**	
16.0- < 17.0	1 (0.03)	13,044 (0.59)	0.07	0.01–0.51	**0.008**	
17.0- < 18.0	1 (0.03)	9,658 (0.44)	0.10	0.01–0.69	**0.020**	
Veterinary Group						**< 0.001**
A	1 (0.03)	2,243 (0.10)	0.41	0.06–2.95	0.378	
B	856 (28.26)	603,744 (26.99)	1.30	1.18–1.42	**< 0.001**	
C	1051 (34.70)	769,575 (34.4)	Reference category			
D	66 (2.18)	36,712 (1.64)	1.65	1.28–2.13	**< 0.001**	
E	473 (15.62)	381,586 (17.06)	1.01	0.90–1.13	0.876	
F	582 (19.21)	443,134 (19.81)	1.00	0.91–1.11	0.951	

Column percentages shown in brackets. P-values < 0.050 are bolded. *CI confidence interval.

#### Entropion.

**Fig 2:**
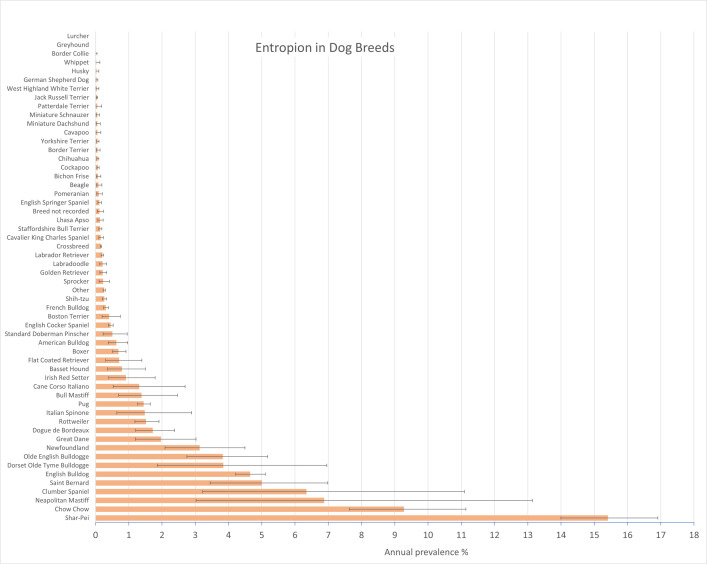
Annual prevalence (%) of entropion in dog breeds under primary veterinary care in the VetCompass Programme in the UK in 2019. The horizontal bars represent 95% confidence intervals.

Of the 3,029 confirmed conformational eyelid disorder cases during 2019, 2,752 (90.86%) included a diagnosis of entropion. These included 67/3,029 (2.21%) conformational eyelid disorder cases that were recorded with both entropion and ectropion. After accounting for the subsampling protocol, the estimated annual prevalence for entropion in dogs overall was 0.33% (95% CI: 0.32–0.34). Among the 2,752 entropion cases, 2,275 were first diagnosed in 2019, giving an annual incidence risk for entropion diagnosis of 0.27% (95% CI 0.26–0.28). Breeds with the highest annual prevalence for entropion were Shar-Pei (15.41%, 95% CI 14.00–16.91), Chow Chow (9.28%, 95% CI 7.64–11.14), Neapolitan Mastiff (6.88%, 95% CI 3.02–13.14), Clumber Spaniel (6.34%, 95% CI 3.22–11.09), Saint Bernard (5.00%, 95% CI 3.44–6.99) and English Bulldog (4.65%, 95% CI 4.22–5.11) (Fig 2).

#### Ectropion.

**Fig 3:**
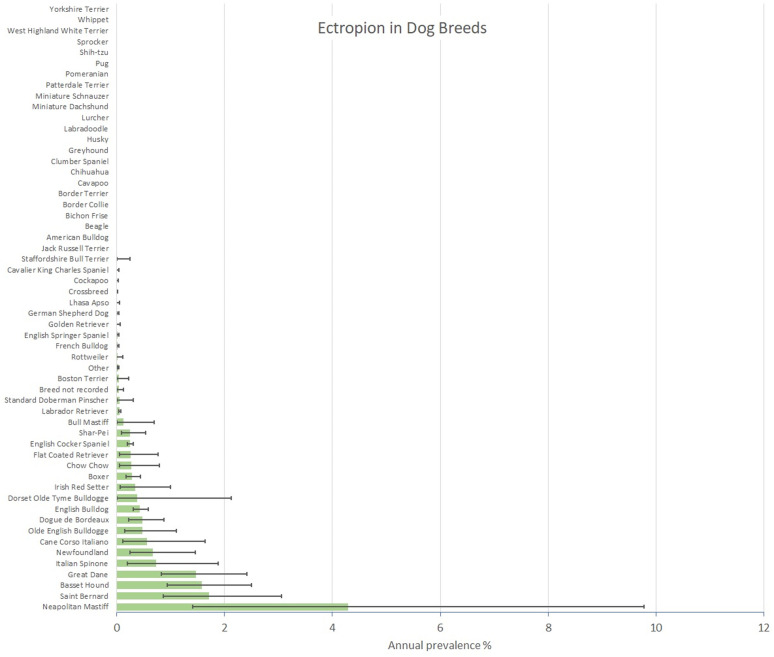
Annual prevalence (%) of ectropion in dog breeds under primary veterinary care in the VetCompass Programme in the UK in 2019. The horizontal bars represent 95% confidence intervals.

Of the 3,029 confirmed conformational eyelid disorder cases during 2019, 344 (11.36%) included a diagnosis of ectropion. After accounting for the subsampling protocol, the estimated annual prevalence for entropion in dogs overall was 0.04% (95% CI: 0.04–0.05). Among the 344 ectropion cases overall, there were 305 that were first diagnosed in 2019, giving an annual incidence risk for ectropion of 0.04% (95% CI 0.03–0.04). Breeds with the highest annual prevalence for ectropion were Neapolitan Mastiff(4.30%, 95% CI 1.41–9.77), Saint Bernard (1.72%, 95% CI 0.86–3.05), Basset Hound (1.59%, 95% CI 0.94–2.49) and Great Dane (1.47%, 95% CI 0.83–2.42) (Fig 3).

### Clinical

#### Entropion.

**Fig 4:**
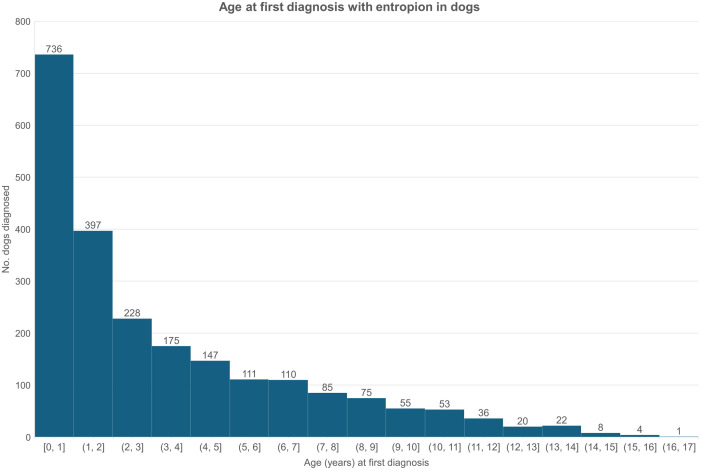
Age at first diagnosis of *entropion* in dogs under primary veterinary care during 2019 at practices collaborating in VetCompass in the UK. n = 2,275.

Among 2,263 of the 2,752 entropion cases with age data available, the median age at first recorded diagnosis in all available EHRs was 2.02 years (IQR 0.69–5.15, range 0.02–16.99) (Fig 4). From 1,875 (82.42%) of cases with the laterality recorded, 1,148 (61.23%) were recorded with bilateral entropion while 385 (20.53%) were unilateral right eye and 342 (18.24%) were unilateral left eye. Among 1,432 cases where information on the eyelid location of entropion was recorded, 1,083 (75.63%) were recorded on the lower eyelid with 294 (20.53%) recorded on the upper eyelid, and 222 (15.50%) were recorded on the medial canthus with 83 (5.80%) recorded on the lateral canthus.

Among 1,540 (67.69%) of entropion cases with at least one clinical sign recorded, the most common clinical signs were ocular discharge (n = 735, 47.73%), epiphora (426, 27.79%), blepharospasm (353, 22.92%) (S1 Table). At least one other ocular condition was recorded as comorbid with entropion in 1,019 (44.79%) cases. Of these, the most common were conjunctivitis (414, 40.63%), corneal ulceration (278, 27.28%) and trichiasis (104, 10.21%) (S2 Table). It should be noted that there was no certainty that these clinical signs or comorbid disorders were definitely caused by the eyelid conformational disorders. Information on the clinical management plan at first diagnosis of entropion was recorded in 1,906 (83.78%) cases. Of these, the most common plans included medical care (1,133, 59.44%) and surgery (1,098, 57.61%) (S3 Table). There was evidence in the available clinical records that surgical management was carried out for 414/2,275 (18.20%) of the entropion cases. Of the 216 (52.17%) surgical entropion cases with information available, the surgical methods used included Celsus-Hotz (86, 39.81%), stay sutures (83, 38.43%) and wedge resection (28, 12.96%) (S4 Table).

#### Ectropion.

**Fig 5:**
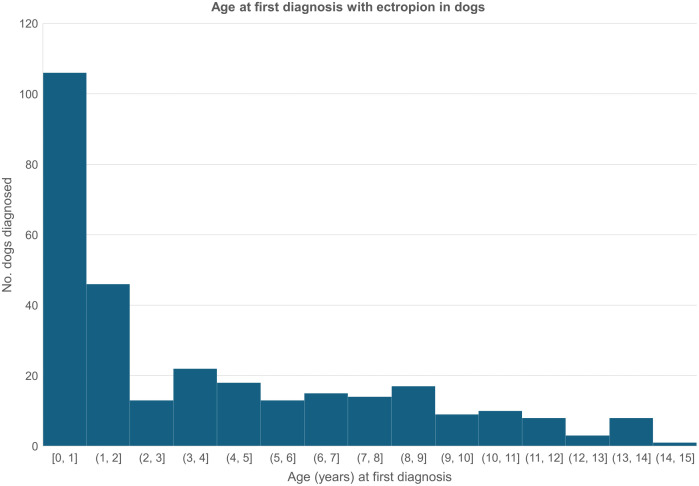
Age at first diagnosis of *ectropion* in dogs under primary veterinary care during 2019 at practices collaborating in VetCompass in the UK. n = 305.

Among 303 of the 344 ectropion cases overall with age data available, the median age at first recorded diagnosis in all available EHRs was 2.15 years (IQR 0.69–6.90, range 0.16–14.94) (Fig 5). From 230 (75.41%) ectropion cases with the laterality recorded, 179 (77.83%) were recorded with bilateral ectropion while 29 (12.61%) were unilateral left eye and 22 (9.57%) were unilateral right eye. Among 141 (46.23%) cases where information on the eyelid location of ectropion was recorded, 133 (94.33%) were recorded on the lower eyelid with 1 (0.71%) recorded on the upper eyelid, and 10 (7.09%) were recorded on the lateral canthus with 2 (1.42%) recorded on the lateral canthus (some had more than one location recorded).

Among 167 (65.75%) of ectropion cases with at least one clinical sign recorded, the most common clinical signs were ocular discharge (n = 87, 52.10%), conjunctival redness/hyperemia (46, 27.54%) and epiphora (25, 14.97%) (S5 Table). At least one other ocular condition was recorded as comorbid with ectropion in 117 (38.36%) cases. Of these, the most common were conjunctivitis (55, 31.07%), diamond eye (17, 9.60%) and corneal ulceration (16, 9.04%) (S6 Table). Information on the clinical management plan at first diagnosis of ectropion was recorded in 201 (65.90%) cases. Of these, the most common plans included medical care (n = 121, 60.20%) and surgery (65, 32.34%) (S7 Table). There was evidence in the available clinical records that surgical management was carried out for 12/305 (3.93%) of the ectropion cases. Of the 8 (66.67%) surgical ectropion cases with information available, the surgical methods used included wedge resection (n = 6, 75.00%), Celsus-Hotz (2, 25.00%), Stades forced granulation procedure (1, 12.50%) and tarsorraphy (1, 12.50%).

### Risk factor analysis

#### Conformational eyelid disorders overall.

All the risk factors assessed (breed, breed purity, Kennel Club recognised breed, Kennel Club breed group, skull conformation, brachycephalic severity, age (years), adult bodyweight, age. ear carriage, coat length, sex-neuter status, spaniel type and veterinary group) were liberally associated with conformational eyelid disorder in univariable logistic regression modelling and were evaluated using multivariable logistic regression modelling. The final breed-focused multivariable model retained three risk factors: breed, age (years) and veterinary group (Table 1). Sex-neuter was not retained in the final model. No biologically significant interactions were identified. McKelvey & Zavoina’s R^2^ value of 0.289 showed that 28.9% of the total variance was explained by the risk factors in the model. The final model showed good discrimination (area under the ROC curve: 0.838). After accounting for the effects of the other variables evaluated in the multivariable modelling, 27 breeds showed increased odds ratio of conformational eyelid disorder compared with general crossbred dogs. The breeds with the highest odds ratios included Shar-Pei (odds ratio [OR] 107.38, 95% CI 92.51–124.64, P < 0.001), Chow Chow (OR 60.05, 95% CI 48.06–75.01, P < 0.001) and Neapolitan Mastiff (OR 58.7, 95% CI 31.77–108.46, P < 0.001). Fifteen breeds showed reduced odds ratios of conformational eyelid disorder compared with general crossbreds. The breeds with the lowest odds ratios included: Border Collie (OR 0.05, 95% CI 0.01–0.21, P < 0.001), Whippet (OR 0.12, 95% CI 0.02–0.83, P = 0.032) and Husky (OR 0.18, 95% CI 0.04–0.70, P = 0.014). No cases were detected in the Greyhound or Lurcher. The odds ratio of having a diagnosis with conformational eyelid disorder were strongly age related, with the highest odds ratio in the 1 to less than 2-year-old age group. The veterinary group attended was retained in the multivariable modelling to account for some residual confounding effects (Table 1).

As described in the methods, breed-derived variables were introduced individually to replace *breed* in the final breed-focused model. Compared with general crossbred dogs, purebred dogs had 2.60 times higher odds ratio (95% CI 2.32–2.91, P-value < 0.001) of conformational eyelid disorder. Kennel Club recognised breeds had 2.47 times the odds ratio compared to breeds not recognised by the Kennel Club (95% CI 2.24–2.71, P < 0.001). Three Kennel Club breed groups (Working, Utility and Gundog) showed higher odds ratio compared to breeds that were not recognised by the Kennel Club, while two breed groups showed lowed odds ratio (Pastoral and Terrier). Breeds with brachycephalic skull conformation (OR 1.74, 95% CI 1.61–1.89, P-value < 0.001) had higher odds ratio compared with breeds with mesocephalic skull conformation, while breeds with a dolichocephalic skull conformation had reduced odds ratio (OR 0.50, 95% CI 0.42–0.49, P-value < 0.001). Among the dogs from breeds with brachycephaly, compared to breeds with mild brachycephaly, breeds with moderate brachycephaly showed reduced odds ratio while breeds with severe brachycephaly showed increased odds ratio. There was a strong trend towards increasing odds ratio as the adult bodyweight increased. Breeds with erect ear carriage had the lowest odds ratio of conformational eyelid disorder. Compared with breeds with medium length coats, breeds with short coat had higher odds ratio and breeds with long coat had reduced odds ratio. Spaniel breeds overall had higher odds ratio of conformational eyelid disorder compared with crossbreed dogs (Table 2).

**Table 2:** Variables that replaced breed in multivariable logistic regression modelling for risk factors associated with *conformational eyelid disorder* during 2019 in dogs under primary veterinary care in the VetCompass™ Programme in the UK.

Risk factor	Case No. (%)	Non-case No. (%)	Odds ratio	95% CI*	Category *P*-value	Variable *P*-value
Breed purity						
General crossbred	350 (11.55)	534,753 (23.90)	Reference category	~		**< 0.001**
Designer crossbreed	62 (2.05)	148,843 (6.65)	0.51	0.38–0.67	**< 0.001**	
Purebred	2,606 (86.03)	1,540,146 (68.85)	2.60	2.32–2.91	**< 0.001**	
Kennel Club recognised breed						**< 0.001**
Not Kennel Club recognised breed	520 (17.17)	723,548 (32.34)	Reference category	~		
Kennel Club recognised breed	2,498 (82.47)	1,500,194 (67.06)	2.47	2.24–2.71	**< 0.001**	
Kennel Club Breed Group						**< 0.001**
Not Kennel Club recognised breed			Reference category	~		
Working	345 (11.39)	76,252 (3.41)	6.34	5.53–7.26	**< 0.001**	
Utility	1,135 (37.47)	260,200 (11.63)	5.84	5.26–6.48	**< 0.001**	
Gundog	523 (17.27)	369,932 (16.54)	2.06	1.82–2.33	**< 0.001**	
Toy	318 (10.50)	277,400 (12.40)	1.65	1.43–1.90	**< 0.001**	
Hound	63 (2.08)	97,174 (4.34)	0.90	0.69–1.17	0.426	
Terrier	93 (3.07)	292,014 (13.05)	0.54	0.43–0.67	**< 0.001**	
Pastoral	21 (0.69)	127,222 (5.69)	0.24	0.16–0.37	**< 0.001**	
Skull conformation						
Mesocephalic	1,349 (44.54)	963,465 (43.07)	Reference category	~		**< 0.001**
Brachycephalic	1,114 (36.78)	391,118 (17.48)	1.74	1.61–1.89	**< 0.001**	
Dolichocephalic	143 (4.72)	185,563 (8.30)	0.50	0.42–0.59	**< 0.001**	
Brachycephalic severity						
Mild	103 (9.16)	46,839 (11.58)	Reference category	~		**< 0.001**
Moderate	149 (13.24)	134,524 (33.27)	0.47	0.36–0.60	**< 0.001**	
Severe	862 (76.62)	209,755 (51.87)	1.67	1.36–2.06	**< 0.001**	
Median adult bodyweight (kg)						
<10 kg	299 (15.15)	532,907 (34.76)	Reference category	~		**< 0.001**
10.0- < 15.0 kg	258 (13.08)	289,068 (18.86)	1.52	1.28–1.79	**< 0.001**	
15.0- < 20.0 kg	227 (11.51)	186,499 (12.17)	2.16	1.82–2.57	**< 0.001**	
20.0- < 25.0 kg	297 (15.05)	158,029 (10.31)	3.57	3.04–4.19	**< 0.001**	
25.0- < 30.0 kg	352 (17.84)	139,523 (9.10)	4.67	4.00–5.46	**< 0.001**	
30.0- < 40.0 kg	318 (16.12)	177,601 (11.59)	3.37	2.88-3.95	**< 0.001**	
40.0- < 50.0 kg	98 (4.97)	38,148 (2.49)	4.82	3.84–6.06	**< 0.001**	
50.0- < 60.0 kg	62 (3.14)	7,514 (0.49)	13.97	10.61–18.39	**< 0.001**	
≥ 60 kg	62 (3.14)	3,616 (0.24)	28.41	21.55–37.45	**< 0.001**	
Ear carriage						
Erect	306 (10.15)	378,005 (17.03)	Reference category	~		**< 0.001**
Semi-erect	1,206 (39.99)	429,511 (19.35)	3.97	3.50–4.51	**< 0.001**	
V-shaped (drop)	465 (15.42)	303,414 (13.67)	2.08	1.80–2.40	**< 0.001**	
Pendulous	627 (20.79)	425,413 (19.16)	1.96	1.71–2.24	**< 0.001**	
General crossbred	412 (13.66)	683,596 (30.79)	0.75	0.65–0.88	**< 0.001**	
Coat length						**< 0.001**
Short	1,856 (61.99)	790,795 (37.05)	1.74	1.58–1.90	**< 0.001**	
Medium	610 (20.37)	474,022 (22.21)	Reference category	~		
Long	116 (3.87)	186,269 (8.73)	0.49	0.40–0.60	**< 0.001**	
General crossbred dogs	412 (13.76)	683,596 (32.02)	0.43	0.38–0.48	**< 0.001**	
Spaniel type						**< 0.001**
General crossbred	350 (11.60)	534,753 (24.05)	Reference category	~		
Non-spaniel type purebred	2,282 (75.61)	1,339,624 (60.24)	2.61	2.33–2.92	**< 0.001**	
Spaniel type purebred	324 (10.74)	200,522 (9.02)	2.53	2.17–2.94	**< 0.001**	
Non-spaniel designer crossbred	28 (0.93)	50,504 (2.27)	0.76	0.51–1.12	0.166	
Spaniel type designer crossbred	34 (1.13)	98,339 (4.42)	0.40	0.28–0.57	**< 0.001**	

Column percentages shown in brackets. P-values < 0.050 are bolded. *CI confidence interval.

#### Entropion.

All the risk factors assessed were liberally associated with entropion in univariable logistic regression modelling and were evaluated using multivariable logistic regression modelling. The final breed-focused multivariable model retained three risk factors: breed, age (years) and veterinary group (Table 3). Sex-neuter was not retained in the final model. No biologically significant interactions were identified. McKelvey & Zavoina’s R^2^ value of 0.274 showed that 27.4% of the total variance was explained by the risk factors in the model. The final model showed good discrimination (area under the ROC curve: 0.834). After accounting for the effects of the other variables evaluated in the multivariable modelling, 28 breeds showed increased odds ratio of entropion compared with general crossbred dogs. The breeds with the highest odds ratio included Shar-Pei (OR 92.48, 95% CI 79.55–107.52, P < 0.001), Chow Chow (OR 53.46, 95% CI 42.66–67.00, P < 0.001) and Neapolitan Mastiff (OR 39.11, 95% CI 19.20–79.68, P < 0.001). Fourteen breeds showed reduced odds ratio of entropion compared with general crossbreds. The breeds with the lowest odds ratio included: Border Collie (OR 0.05, 95% CI 0.01–0.22, P < 0.001), Whippet (OR 0.12, 95% CI 0.02–0.88, P = 0.036) and Husky (OR 0.18, 95% CI 0.05–0.73, P = 0.017). No cases were detected in the Greyhound or Lurcher. The odds ratio of having a diagnosis with entropion were strongly age related, with the highest odds ratio in the 1 to less than 2-year-old age group. The veterinary group attended was retained in the multivariable modelling to account for some residual confounding effects (Table 3).

**Table 3:** Descriptive and breed-focused multivariable logistic regression results for risk factors associated with *entropion* cases during 2019 in dogs under primary veterinary care in the VetCompass™ Programme in the UK.

Variable	Case No. (%)	Non-case No. (%)	Odds Ratio	95% CI	Category *P*-value	Variable *P*-value
Breed						**< 0.001**
Crossbreed	334 (12.14)	536001 (23.85)	Reference category			
Shar-Pei	375 (13.63)	6171 (0.27)	92.48	79.55–107.52	**< 0.001**	
Chow Chow	103 (3.74)	2693 (0.12)	53.46	42.66–67	**< 0.001**	
Neapolitan Mastiff	8 (0.29)	300 (0.01)	39.11	19.2–79.68	**< 0.001**	
Clumber Spaniel	11 (0.40)	534 (0.02)	32.93	17.93–60.5	**< 0.001**	
Saint Bernard	32 (1.16)	1434 (0.06)	31.56	21.86–45.57	**< 0.001**	
English Bulldog	409 (14.86)	22614 (1.01)	25.21	21.76–29.2	**< 0.001**	
Dorset Olde Tyme Bulldogge	10 (0.36)	684 (0.03)	20.41	10.82–38.51	**< 0.001**	
Newfoundland	28 (1.02)	2146 (0.10)	19.90	13.49–29.35	**< 0.001**	
Olde English Bulldogge	40 (1.45)	3058 (0.14)	17.79	12.76–24.81	**< 0.001**	
Italian Spinone	8 (0.29)	954 (0.04)	13.17	6.51–26.64	**< 0.001**	
Great Dane	20 (0.73)	2603 (0.12)	11.44	7.27–18	**< 0.001**	
Dogue de Bordeaux	36 (1.31)	5120 (0.23)	10.30	7.29–14.56	**< 0.001**	
Bull Mastiff	11 (0.40)	1791 (0.08)	10.08	5.52–18.44	**< 0.001**	
Rottweiler	71 (2.58)	13132 (0.58)	8.32	6.43–10.75	**< 0.001**	
Pug	229 (8.32)	40280 (1.79)	8.27	6.98–9.8	**< 0.001**	
Irish Red Setter	8 (0.29)	2145 (0.10)	6.20	3.07–12.53	**< 0.001**	
Cane Corso Italiano	7 (0.25)	1840 (0.08)	5.05	2.38–10.7	**< 0.001**	
Flat Coated Retriever	8 (0.29)	2670 (0.12)	4.72	2.34–9.54	**< 0.001**	
Boxer	48 (1.74)	17524 (0.78)	4.54	3.35–6.14	**< 0.001**	
Basset Hound	9 (0.33)	3929 (0.17)	4.06	2.09–7.87	**< 0.001**	
American Bulldog	20 (0.73)	8613 (0.38)	3.30	2.1–5.19	**< 0.001**	
English Cocker Spaniel	162 (5.89)	96662 (4.30)	2.64	2.18–3.18	**< 0.001**	
Boston Terrier	10 (0.36)	5456 (0.24)	2.60	1.38–4.88	**0.003**	
Standard Doberman Pinscher	9 (0.33)	5727 (0.25)	2.35	1.21–4.56	**0.012**	
Sprocker	10 (0.36)	9338 (0.42)	1.62	0.86–3.05	0.132	
Shih-tzu	65 (2.36)	67303 (2.99)	1.57	1.2–2.05	**0.001**	
Other purebreeds	216 (7.85)	230270 (10.24)	1.48	1.25–1.76	**< 0.001**	
French Bulldog	71 (2.58)	66926 (2.98)	1.38	1.07–1.79	**0.014**	
Labradoodle	19 (0.69)	21783 (0.97)	1.26	0.78–2.03	0.338	
Labrador Retriever	111 (4.03)	154111 (6.86)	1.19	0.96–1.47	0.119	
Cavalier King Charles Spaniel	22 (0.80)	35218 (1.57)	1.07	0.7–1.65	0.750	
Golden Retriever	19 (0.69)	27472 (1.22)	1.05	0.65–1.68	0.855	
Breed not recorded	8 (0.29)	13304 (0.59)	0.95	0.42–2.14	0.895	
Lhasa Apso	13 (0.47)	24540 (1.09)	0.92	0.53–1.61	0.783	
Staffordshire Bull Terrier	41 (1.49)	93842 (4.18)	0.77	0.56–1.07	0.122	
Pomeranian	7 (0.25)	14837 (0.66)	0.70	0.33–1.48	0.350	
English Springer Spaniel	22 (0.80)	51780 (2.30)	0.69	0.44–1.07	0.097	
Yorkshire Terrier	14 (0.51)	53232 (2.37)	0.47	0.27–0.79	**0.005**	
Beagle	6 (0.22)	20223 (0.90)	0.46	0.21–1.04	0.062	
Border Terrier	6 (0.22)	24691 (1.10)	0.43	0.19–0.96	**0.040**	
Bichon Frise	6 (0.22)	25156 (1.12)	0.42	0.19–0.93	**0.033**	
Chihuahua	22 (0.80)	80587 (3.59)	0.42	0.27–0.64	**< 0.001**	
Miniature Schnauzer	5 (0.18)	21149 (0.94)	0.38	0.16–0.91	**0.031**	
Cockapoo	20 (0.73)	73017 (3.25)	0.35	0.22–0.56	**< 0.001**	
Jack Russell Terrier	19 (0.69)	101275 (4.51)	0.35	0.22–0.56	**< 0.001**	
Patterdale Terrier	2 (0.07)	10759 (0.48)	0.32	0.08–1.28	0.106	
Cavapoo	3 (0.11)	14143 (0.63)	0.30	0.1–0.93	**0.037**	
Miniature Dachshund	4 (0.15)	24827 (1.10)	0.23	0.09–0.62	**0.004**	
West Highland White Terrier	4 (0.15)	35810 (1.59)	0.22	0.08–0.58	**0.002**	
German Shepherd Dog	6 (0.22)	47401 (2.11)	0.20	0.09–0.44	**< 0.001**	
Husky	2 (0.07)	17335 (0.77)	0.18	0.05–0.73	**0.017**	
Whippet	1 (0.04)	12764 (0.57)	0.12	0.02–0.88	**0.036**	
Border Collie	2 (0.07)	61800 (2.75)	0.05	0.01–0.22	**< 0.001**	
Greyhound	0 (0.00)	12640 (0.56)				
Lurcher	0 (0.00)	16051 (0.71)				
Age (years)						**< 0.001**
<1.0	354 (12.91)	232385 (10.42)	0.68	0.6–0.78	**< 0.001**	
1.0- < 2.0	627 (22.87)	267086 (11.98)	Reference category			
2.0- < 3.0	423 (15.43)	214129 (9.60)	0.91	0.8–1.03	0.141	
3.0- < 4.0	243 (8.86)	184069 (8.25)	0.63	0.54–0.73	**< 0.001**	
4.0- < 5.0	227 (8.28)	178229 (7.99)	0.64	0.55–0.75	**< 0.001**	
5.0- < 6.0	158 (5.76)	164082 (7.36)	0.50	0.42–0.6	**< 0.001**	
6.0- < 7.0	162 (5.91)	155077 (6.95)	0.57	0.48–0.68	**< 0.001**	
7.0- < 8.0	130 (4.74)	144643 (6.49)	0.52	0.43–0.63	**< 0.001**	
8.0- < 9.0	105 (3.83)	135151 (6.06)	0.46	0.37–0.57	**< 0.001**	
9.0- < 10.0	91 (3.32)	121730 (5.46)	0.46	0.37–0.57	**< 0.001**	
10.0- < 11.0	79 (2.88)	108587 (4.87)	0.48	0.38–0.61	**< 0.001**	
11.0- < 12.0	46 (1.68)	93441 (4.19)	0.34	0.25–0.46	**< 0.001**	
12.0- < 13.0	40 (1.46)	79169 (3.55)	0.38	0.28–0.53	**< 0.001**	
13.0- < 14.0	23 (0.84)	60942 (2.73)	0.31	0.2–0.47	**< 0.001**	
14.0- < 15.0	19 (0.69)	42712 (1.92)	0.40	0.25–0.63	**< 0.001**	
15.0- < 16.0	13 (0.47)	25938 (1.16)	0.49	0.28–0.85	**0.012**	
16.0- < 17.0	1 (0.04)	13062 (0.59)	0.08	0.01–0.59	**0.013**	
17.0- < 18.0	1 (0.04)	9667 (0.43)	0.11	0.02–0.8	**0.029**	
Veterinary Group						
A	1 (0.04)	2256 (0.10)	0.44	0.06–3.16	0.416	
B	766 (27.83)	606973 (27.00)	1.27	1.15–1.4	**< 0.001**	
C	970 (35.25)	773144 (34.40)	Reference category			**< 0.001**
D	53 (1.93)	36946 (1.64)	1.45	1.09–1.92	**0.010**	
E	414 (15.04)	383370 (17.06)	0.98	0.87–1.1	0.705	
F	548 (19.91)	444976 (19.80)	1.03	0.92–1.14	0.610	

Column percentages shown in brackets. P-values < 0.050 are bolded. *CI confidence interval.

After introducing breed-derived variables individually to replace *breed* in the final breed-focused model, compared with general crossbred dogs, purebred dogs had 2.45 times higher odds ratio (95% CI 2.18–2.75, P-value < 0.001) of entropion. Kennel Club recognised breeds had 2.33 times the odds ratio compared to breeds not recognised by the Kennel Club (95% CI 2.11–2.56, P < 0.001). Four Kennel Club breed groups (Utility, Working, Toy and Gundog) showed higher odds ratio compared to breeds that were not recognised by the Kennel Club, while three breed groups showed lower odds ratio (Pastoral, Terrier and Hound). Breeds with brachycephalic skull conformation (OR 1.83, 95% CI 1.69–2.00, P-value < 0.001) had higher odds ratio compared with breeds with mesocephalic skull conformation, while breeds with a dolichocephalic skull conformation had reduced odds ratio (OR 0.42, 95% CI 0.34–0.51, P-value < 0.001). Among the dogs from breeds with brachycephaly, compared to breeds with mild brachycephaly, breeds with moderate brachycephaly showed reduced odds ratio while breeds with severe brachycephaly showed increased odds ratio. There was a strong trend towards increasing odds ratio as the adult bodyweight increased. Breeds with erect ear carriage had the lowest odds ratio of entropion. Compared with breeds with medium length coats, breeds with short coat had higher odds ratio and breeds with long coat had reduced odds ratio. Spaniel breeds overall had higher odds ratio of entropion compared with crossbreed dogs (Table 4).

**Table 4:** Variables that replaced breed in multivariable logistic regression modelling for risk factors associated with *entropion* during 2019 in dogs under primary veterinary care in the VetCompass™ Programme in the UK.

Risk factor	Case No. (%)	Non-case No. (%)	Odds ratio	95% CI*	Category *P*-value	Variable *P*-value
Breed purity						
General crossbred	334 (12.14)	536,001 (23.85)	Reference category	~		**< 0.001**
Designer crossbreed	61 (2.22)	149,247 (6.64)	0.52	0.39–0.68	**< 0.001**	
Purebred	2,349 (85.36)	1,549,113 (68.92)	2.45	2.18–2.75	**< 0.001**	
Kennel Club recognised breed						**< 0.001**
Not Kennel Club recognised breed	496 (18.02)	725,598 (32.28)	Reference category	~		
Kennel Club recognised breed	2,248 (81.69)	1,508,763 (67.13)	2.33	2.11–2.56	**< 0.001**	
Kennel Club Breed Group						**< 0.001**
Not Kennel Club recognised breed	496 (18.02)	725,598 (32.28)	Reference category			
Utility	1097 (39.86)	263,652 (11.73)	5.83	5.24–6.49	**< 0.001**	
Working	295 (10.72)	77,228 (3.44)	5.62	4.86–6.49	**< 0.001**	
Toy	317 (11.52)	278,408 (12.39)	1.72	1.49–1.98	**< 0.001**	
Gundog	393 (14.28)	372,035 (16.55)	1.62	1.42–1.85	**< 0.001**	
Hound	38 (1.38)	97,488 (4.34)	0.57	0.41–0.79	**0.001**	
Terrier	90 (3.27)	292,548 (13.02)	0.55	0.44–0.69	**< 0.001**	
Pastoral	18 (0.65)	127,404 (5.67)	0.22	0.14–0.35	**< 0.001**	
Skull conformation						
Mesocephalic	1,192 (43.31)	968,211 (43.08)	Reference category	~		**< 0.001**
Brachycephalic	1,052 (38.23)	394,687 (17.56)	1.83	1.69–2.00	**< 0.001**	
Dolichocephalic	105 (3.82)	186,215 (8.28)	0.42	0.34–0.51	**< 0.001**	
Brachycephalic severity						
Mild	84 (7.92)	47,229 (11.58)	Reference category	~		**< 0.001**
Moderate	136 (12.83)	135,018 (33.09)	0.53	0.40–0.70	**< 0.001**	
Severe	832 (78.49)	212,440 (52.07)	1.98	1.58–2.49	**< 0.001**	
Median adult bodyweight (kg)						
<10 kg	298 (16.60)	534,232 (34.69)	Reference category	~		**< 0.001**
10.0- < 15.0 kg	226 (12.59)	290,252 (18.85)	1.33	1.12–1.58	**0.001**	
15.0- < 20.0 kg	195 (10.86)	187,367 (12.17)	1.87	1.56–2.24	**< 0.001**	
20.0- < 25.0 kg	279 (15.54)	159,054 (10.33)	3.37	2.86–3.96	**< 0.001**	
25.0- < 30.0 kg	324 (18.05)	140,561 (9.13)	4.32	3.69–5.05	**< 0.001**	
30.0- < 40.0 kg	281 (15.65)	178,731 (11.61)	3.00	2.55–3.53	**< 0.001**	
40.0- < 50.0 kg	84 (4.68)	38,445 (2.50)	4.14	3.25–5.28	**< 0.001**	
50.0- < 60.0 kg	55 (3.06)	7,702 (0.50)	12.09	9.06–16.15	**< 0.001**	
≥ 60 kg	53 (2.95)	3,764 (0.24)	23.29	17.34–31.27	**< 0.001**	
Ear carriage						
Erect	294 (10.72)	379,287 (17.00)	Reference category	~		**< 0.001**
Semi-erect	1,173 (42.78)	433,100 (19.42)	4.02	3.53–4.57	**< 0.001**	
V-shaped (drop)	397 (14.48)	305,078 (13.68)	1.85	1.59–2.15	**< 0.001**	
Pendulous	483 (17.61)	427,841 (19.18)	1.57	1.36–1.82	**< 0.001**	
General crossbred	395 (14.41)	685,248 (30.72)	0.76	0.65–0.88	**< 0.001**	
Coat length						**< 0.001**
Short	1,713 (62.98)	796,713 (37.14)	1.94	1.76–2.15	**< 0.001**	
Medium	500 (18.38)	476,383 (22.21)	Reference category	~		
Long	112 (4.12)	186,822 (8.71)	0.57	0.47–0.70	**< 0.001**	
General crossbred dogs	395 (14.52)	685,248 (31.94)	0.50	0.44–0.57	**< 0.001**	
Spaniel type						**< 0.001**
General crossbred	334 (12.17)	536,001 (23.99)	Reference category	~		
Non-spaniel type purebred	2,113 (77.00)	1,347,334 (60.30)	2.52	2.25–2.83	**< 0.001**	
Spaniel type purebred	236 (8.60)	201,779 (9.03)	1.92	1.63–2.27	**< 0.001**	
Non-spaniel designer crossbred	28 (1.02)	50,640 (2.27)	0.79	0.54–1.18	0.250	
Spaniel type designer crossbred	33 (1.20)	98,607 (4.41)	0.40	0.28–0.58	**< 0.001**	

Column percentages shown in brackets. P-values < 0.050 are bolded. *CI confidence interval.

#### Ectropion.

All the risk factors assessed were liberally associated with ectropion in univariable logistic regression modelling and were evaluated using multivariable logistic regression modelling. The final breed-focused multivariable model retained three risk factors: breed, age (years) and veterinary group (Table 5). Sex-neuter was not retained in the final model. No biologically significant interactions were identified. McKelvey & Zavoina’s R^2^ value of 0.359 showed that 35.9% of the total variance was explained by the risk factors in the model. The final model showed good discrimination (area under the ROC curve: 0.886). After accounting for the effects of the other variables evaluated in the multivariable modelling, 19 breeds showed increased odds ratio of ectropion compared with general crossbred dogs. The breeds with the highest odds ratio included Neapolitan Mastiff (OR 426.97, 95% CI 156.82–1162.50, P < 0.001), Saint Bernard (OR 200.71, 95% CI 94.36–426.92, P < 0.001), Great Dane (OR 153.4, 95% CI 77.05–305.41, P < 0.001) and Basset Hound (OR 147.09, 95% CI 76.32–283.48, P < 0.001). No breeds showed statistically reduced odds ratio of ectropion compared with general crossbreds but there were 22 breeds that did not have any cases of ectropion identified. The odds ratio of having a diagnosis with ectropion were strongly age related, with the highest odds ratio in the 1 to less than 2-year-old age group. The veterinary group attended was retained in the multivariable modelling to account for some residual confounding effects (Table 5).

**Table 5:** Descriptive and breed-focused multivariable logistic regression results for risk factors associated with *ectropion* cases during 2019 in dogs under primary veterinary care in the VetCompass™ Programme in the UK.

Risk factor	Case No. (%)	Non-case No. (%)	Odds ratio	95% CI*	Category *P*-value	Variable *P*-value
Breed						**< 0.001**
Crossbreed	19 (5.52)	536316 (23.84)	Reference category			
Neapolitan Mastiff	5 (1.45)	303 (0.01)	426.97	156.82–1162.50	**< 0.001**	
Saint Bernard	11 (3.20)	1455 (0.06)	200.71	94.36–426.92	**< 0.001**	
Great Dane	15 (4.36)	2608 (0.12)	153.40	77.05–305.41	**< 0.001**	
Basset Hound	18 (5.23)	3920 (0.17)	147.09	76.32–283.48	**< 0.001**	
Italian Spinone	4 (1.16)	958 (0.04)	113.80	38.38–337.44	**< 0.001**	
Newfoundland	6 (1.74)	2168 (0.10)	77.88	30.85–196.62	**< 0.001**	
Dogue de Bordeaux	10 (2.91)	5146 (0.23)	53.86	24.79–117.02	**< 0.001**	
Olde English Bulldogge	5 (1.45)	3093 (0.14)	43.78	16.13–118.85	**< 0.001**	
English Bulldog	38 (11.05)	22985 (1.02)	43.40	24.66–76.37	**< 0.001**	
Irish Red Setter	3 (0.87)	2150 (0.10)	40.56	11.93–137.94	**< 0.001**	
Dorset Olde Tyme Bulldogge	1 (0.29)	693 (0.03)	40.48	5.38–304.59	**< 0.001**	
Cane Corso Italiano	3 (0.87)	1844 (0.08)	39.63	11.59–135.49	**< 0.001**	
Boxer	20 (5.81)	17552 (0.78)	33.79	17.85–63.96	**< 0.001**	
Flat Coated Retriever	3 (0.87)	2675 (0.12)	29.88	8.78–101.68	**< 0.001**	
Chow Chow	3 (0.87)	2793 (0.12)	27.86	8.19–94.85	**< 0.001**	
Chinese Shar-Pei	6 (1.74)	6540 (0.29)	26.34	10.44–66.45	**< 0.001**	
English Cocker Spaniel	90 (26.16)	96734 (4.3)	26.32	15.86–43.69	**< 0.001**	
Bull Mastiff	1 (0.29)	1801 (0.08)	17.05	2.27–128.09	**0.006**	
Labrador Retriever	32 (9.30)	154190 (6.85)	5.99	3.36–10.68	**< 0.001**	
Breed not recorded	3 (0.87)	13309 (0.59)	5.91	1.69–20.71	**0.006**	
Boston Terrier	1 (0.29)	5465 (0.24)	4.84	0.65–36.32	0.125	
Standard Doberman Pinscher	1 (0.29)	5735 (0.25)	4.77	0.64–35.77	0.128	
Other purebred	28 (8.14)	230458 (10.24)	3.33	1.84–6.03	**< 0.001**	
Rottweiler	1 (0.29)	13202 (0.59)	2.16	0.29–16.19	0.454	
English Springer Spaniel	3 (0.87)	51799 (2.30)	1.71	0.50–5.82	0.389	
French Bulldog	4 (1.16)	66993 (2.98)	1.51	0.51–4.48	0.458	
Lhasa Apso	1 (0.29)	24552 (1.09)	1.33	0.18–9.95	0.783	
German Shepherd Dog	2 (0.58)	47405 (2.11)	1.18	0.27–5.09	0.824	
Golden Retriever	1 (0.29)	27490 (1.22)	1.00	0.13–7.46	0.997	
Cavalier King Charles Spaniel	1 (0.29)	35239 (1.57)	0.86	0.11–6.43	0.881	
Staffordshire Bull Terrier	2 (0.58)	93881 (4.17)	0.66	0.15–2.83	0.573	
Cockapoo	2 (0.58)	73035 (3.25)	0.36	0.05–2.66	0.314	
Jack Russell Terrier	1 (0.29)	101293 (4.50)	0.31	0.04–2.33	0.256	
American Bulldog	0 (0.00)	8633 (0.38)	~			
Beagle	0 (0.00)	20229 (0.90)	~			
Bichon Frise	0 (0.00)	25162 (1.12)	~			
Border Collie	0 (0.00)	61802 (2.75)	~			
Border Terrier	0 (0.00)	24697 (1.10)	~			
Cavapoo	0 (0.00)	14146 (0.63)	~			
Chihuahua	0 (0.00)	80609 (3.58)	~			
Clumber Spaniel	0 (0.00)	545 (0.02)	~			
Greyhound	0 (0.00)	12640 (0.56)	~			
Husky	0 (0.00)	17337 (0.77)	~			
Labradoodle	0 (0.00)	21802 (0.97)	~			
Lurcher	0 (0.00)	16051 (0.71)	~			
Miniature Dachshund	0 (0.00)	24831 (1.10)	~			
Miniature Schnauzer	0 (0.00)	21154 (0.94)	~			
Patterdale Terrier	0 (0.00)	10761 (0.48)	~			
Pomeranian	0 (0.00)	14844 (0.66)	~			
Pug	0 (0.00)	40509 (1.80)	~			
Shih-tzu	0 (0.00)	67368 (2.99)	~			
Sprocker	0 (0.00)	9348 (0.42)	~			
West Highland White Terrier	0 (0.00)	35814 (1.59)	~			
Whippet	0 (0.00)	12765 (0.57)	~			
Yorkshire Terrier	0 (0.00)	53246 (2.37)	~			
Age (years)						**< 0.001**
<1.0	48 (14.04)	232691 (10.42)	0.70	0.49–1.00	0.051	
1.0- < 2.0	80 (23.39)	267633 (11.99)	Reference category			
2.0- < 3.0	30 (8.77)	214522 (9.61)	0.51	0.33–0.77	**0.002**	
3.0- < 4.0	27 (7.89)	184285 (8.25)	0.54	0.35–0.83	**0.005**	
4.0- < 5.0	25 (7.31)	178431 (7.99)	0.51	0.33–0.80	**0.004**	
5.0- < 6.0	16 (4.68)	164224 (7.36)	0.35	0.21–0.61	**0.000**	
6.0- < 7.0	18 (5.26)	155221 (6.95)	0.42	0.25–0.71	**0.001**	
7.0- < 8.0	20 (5.85)	144753 (6.48)	0.50	0.31–0.82	**0.006**	
8.0- < 9.0	22 (6.43)	135234 (6.06)	0.59	0.37–0.95	**0.030**	
9.0- < 10.0	11 (3.22)	121810 (5.46)	0.33	0.17–0.62	**0.001**	
10.0- < 11.0	15 (4.39)	108651 (4.87)	0.53	0.30–0.93	**0.026**	
11.0- < 12.0	9 (2.63)	93478 (4.19)	0.37	0.18–0.73	**0.005**	
12.0- < 13.0	7 (2.05)	79202 (3.55)	0.37	0.17–0.8	**0.012**	
13.0- < 14.0	5 (1.46)	60960 (2.73)	0.36	0.15–0.9	**0.029**	
14.0- < 15.0	6 (1.75)	42725 (1.91)	0.72	0.31–1.68	0.452	
15.0- < 16.0	3 (0.88)	25948 (1.16)	0.74	0.23–2.37	0.617	
16.0- < 17.0	0 (0.00)	13063 (0.59)	~			
17.0- < 18.0	0 (0.00)	9668 (0.43)	~			
Veterinary Group						**< 0.001**
A	0 (0.00)	2257 (0.10)	~			
B	111 (32.27)	607628 (27.00)	1.53	1.16–2.02	**0.003**	
C	98 (28.49)	774016 (34.40)	Reference category			
D	14 (4.07)	36985 (1.64)	3.30	1.86–5.88	**< 0.001**	
E	75 (21.8)	383709 (17.05)	1.48	1.09–2.01	**0.012**	
F	46 (13.37)	445478 (19.80)	0.86	0.61–1.23	0.414	

Column percentages shown in brackets. P-values < 0.050 are bolded. *CI confidence interval.

After introducing breed-derived variables individually to replace *breed* in the final breed-focused model, compared with general crossbred dogs, purebred dogs had 5.95 times higher odds ratio (95% CI 3.70–9.57, P-value < 0.001) of ectropion. Kennel Club recognised breeds had 4.69 times the odds ratio compared to breeds not recognised by the Kennel Club (95% CI 3.25–6.75, P < 0.001). Four Kennel Club breed groups (Working, Gundog, Hound and Utility) showed higher odds ratios compared to breeds that were not recognised by the Kennel Club, while two breed groups showed lower odds ratios (Terrier and Toy). Skull conformation overall was not associated with the odds ratio of ectropion. The odds ratio of ectropion rose as adult bodyweight increased. Breeds with erect ear carriage had the lowest odds ratio of ectropion. Compared with breeds with medium length coats, breeds with long coat had reduced odds ratio. Spaniel breeds overall had higher odds ratio of ectropion compared with crossbreed dogs (Table 6).

**Table 6:** Variables that replaced breed in multivariable logistic regression modelling associated with risk factors for *ectropion* during 2019 in dogs under primary veterinary care in the VetCompass™ Programme in the UK.

Risk factor	Case No. (%)	Non-case No. (%)	Odds ratio	95% CI*	Category *P*-value	Variable *P*-value
Breed purity						
General crossbred	19 (5.52)	536,316 (23.84)	Reference category	~		**< 0.001**
Designer crossbreed	2 (0.58)	149,306 (6.64)	0.17	0.02–1.25	0.082	
Purebred	320 (93.02)	1,551,142 (68.94)	5.95	3.70–9.57	**< 0.001**	
Kennel Club recognised breed						**< 0.001**
Not Kennel Club recognised breed	34 (9.88)	726,060 (32.27)	Reference category	~		
Kennel Club recognised breed	307 (89.24)	1,510,704 (67.14)	4.69	3.25–6.75	**< 0.001**	
Kennel Club Breed Group						**< 0.001**
Not Kennel Club recognised breed	34 (9.88)	726,060 (32.27)	Reference category			
Working	76 (22.09)	77,447 (3.44)	21.89	14.48–33.11	**< 0.001**	
Gundog	142 (41.28)	372,286 (16.55)	8.59	5.84 −12.62	**< 0.001**	
Hound	26 (7.56)	97,500 (4.33)	5.81	3.46–9.75	**< 0.001**	
Utility	56 (16.28)	264,693 (11.76)	4.61	2.98–7.12	**< 0.001**	
Pastoral	3 (0.87)	127,419 (5.66)	0.53	0.16–1.75	0.300	
Terrier	3 (0.87)	292,635 (13.01)	0.26	0.08–0.85	0.025	
Toy	1 (0.29)	278,724 (12.39)	0.08	0.01–0.61	**0.014**	
Skull conformation						
Mesocephalic	180 (56.25)	969,223 (62.48)	Reference category	~		0.268
Brachycephalic	96 (30.00)	395,643 (25.51)	1.22	0.95–1.57	0.127	
Dolichocephalic	44 (13.75)	186,276 (12.01)	1.18	0.85–1.64	0.332	
Median adult bodyweight (kg)						**< 0.001**
<10 kg	1 (0.44)	534,529 (34.67)	0.02	0–0.12	**< 0.001**	
10.0- < 15.0 kg	34 (15.11)	290,444 (18.84)	Reference category			
15.0- < 20.0 kg	36 (16.00)	187,526 (12.16)	1.68	1.05–2.69	0.030	
20.0- < 25.0 kg	23 (10.22)	159,310 (10.33)	1.32	0.77–2.24	0.309	
25.0- < 30.0 kg	38 (16.89)	140,847 (9.14)	2.39	1.51–3.81	**< 0.001**	
30.0- < 40.0 kg	48 (21.33)	178,964 (11.61)	2.41	1.55–3.75	**< 0.001**	
40.0- < 50.0 kg	17 (7.56)	38,512 (2.50)	4.01	2.24–7.19	**< 0.001**	
50.0- < 60.0 kg	11 (4.89)	7,746 (0.50)	12.04	6.09–23.79	**< 0.001**	
≥ 60 kg	17 (7.56)	3,800 (0.25)	37.09	20.66–66.59	**< 0.001**	
Ear carriage						
Erect	13 (3.81)	379,568 (17.00)	Reference category	~		**< 0.001**
Semi-erect	56 (16.42)	434,217 (19.45)	4.10	2.24–7.50	**< 0.001**	
V-shaped (drop)	89 (26.10)	305,386 (13.68)	8.82	4.92–15.79	**< 0.001**	
Pendulous	162 (47.51)	428,162 (19.17)	11.43	6.49–20.12	**< 0.001**	
General crossbred	21 (6.16)	685,622 (30.70)	0.83	0.41–1.68	0.608	
Coat length						**< 0.001**
Short	196 (57.48)	798,230 (37.17)	0.95	0.76–1.19	0.665	
Medium	120 (35.19)	476,763 (22.20)	Reference category			
Long	4 (1.17)	186,930 (8.70)	0.09	0.03–0.24	**< 0.001**	
General crossbred dogs	21 (6.16)	685,622 (31.93)	0.11	0.07–0.17	**< 0.001**	
Spaniel type						**< 0.001**
General crossbred	19 (5.57)	536,316 (23.98)	Reference category	~		
Non-spaniel type purebred	224 (65.69)	1,349,223 (60.32)	4.78	2.96–7.73	**< 0.001**	
Spaniel type purebred	96 (28.15)	201,919 (9.03)	13.67	8.26–22.64	**< 0.001**	
Non-spaniel designer crossbred	0 (0.00)	50,668 (2.27)	~			
Spaniel type designer crossbred	2 (0.59)	98,638 (4.41)	0.24	0.03–1.82	**0.168**	

Column percentages shown in brackets. P-values < 0.050 are bolded. *CI confidence interval.

## Discussion

This study is the first to use a national multi-practice dataset of dogs under primary veterinary care to report the prevalence and risk factors of conformational eyelid disorders. The study therefore offers several novel insights compared to previous referral practice population studies. Ophthalmological disorders overall have previously been identified significantly more frequently in purebred dogs than in crossbreed dogs [44] and the current study supports this purebred health disadvantage by also now reporting higher odds ratio for conformational eyelid disorders in purebred dogs. The current results offer further evidence that this purebred predisposition is heavily driven by a limited set of high profile breeds that account for many these cases. The study also supports the value of current international efforts to shift humanity away from normalising extreme conformation in dogs and instead moving towards recognising these extreme conformations as disorders in their own right [26].

Clinical diagnosis of conformational eyelid abnormality is usually straightforward for primary care practitioners [2]. However, achieving the optimal surgical therapy necessary for some of the more complex cases may require specialist care because understanding the subtle interactions and function between the delicate eyelid structures as well as identifying and finding the solution for the inciting cause can be challenging [2]. Nevertheless, the current data confirm that many cases of conformational eyelid disorder are managed under primary care either without surgery or with surgical therapy undertaken at the primary care clinic. Therefore, some previous studies based solely on referral data may have underestimated the overall prevalence of conformational eyelid disorders or may show referral bias towards the more complicated cases and associated breeds [9,11,45]. On the other hand, even despite the large prevalence recorded, the current data may still have heavily underestimated the true prevalence. This is because normalisation of conformational eyelid disorders as “normal for breed” based on currently published breeding standards and breed norms may have led many owners and veterinarians to fail to recognise true cases not presenting with marked or disabling level of severity. While more recently graduated veterinary surgeons receive increasing levels of undergraduate education on conformational and breed-related disorders, this may hold less true for the wider working profession and may therefore result in substantial recording bias on what is considered normal and what is considered a disorder [46]. Conformational eyelid disorder cases only mildly affected with ectropion may show minimal clinical signs or the signs may be controlled with appropriate management by the owner [2]. This may increase the propensity of veterinary surgeons to miss the diagnosis or simply omit acknowledgement of the condition in the clinical records.

In the current study, the annual prevalence of entropion diagnosis (0.33%) was almost tenfold higher in comparison to ectropion (0.04%). While irritating or frankly painful comorbidities such as conjunctivitis and corneal ulceration were recorded to frequently accompany both entropion and ectropion diagnoses, this raises the suspicion that many true cases of entropion and ectropion that were not yet accompanied by severe clinical signs were not recorded. Of the conformational eyelid disorder cases with an accompanying clinical sign recorded, a striking 27% of dogs with entropion and 9% of ectropion cases were co-morbidly diagnosed with concurrent corneal ulceration, a painful and potentially globe-threatening condition [10]. It was also possible, however, that some of these recorded clinical signs and comorbid disorders were not directly associated with the eyelid conformational disorders.

Shar Pei, Chow Chow and Neapolitan Mastiff showed both the highest breed annual prevalence for a conformational eyelid disorder overall and the highest odds ratios for entropion diagnosis. Neapolitan Mastiff, Great Dane and Saint Bernard had the highest odds ratios for ectropion. A high prevalence of both ectropion and entropion in Neapolitan Mastiff is not a new finding, with a study of 152 Neapolitan Mastiffs reporting 24.3% entropion and 23.4% ectropion [47]. The results for the breeds in the current study with the highest prevalence for entropion also reflect recent data from eye tests performed for breeding purposes in the US where Shar Pei, Chow Chow and Clumber Spaniel were diagnosed the most [7]. Regarding ectropion, Neapolitan Mastiff, Great Dane and Clumber Spaniel were breeds most commonly diagnosed in the US data although the prevalence in the Neapolitan Mastiff (51.2%) was substantially higher than for the Great Dane (4.1%) [7]. However, residual differences between breeds across these studies highlight the importance of wide range data collection from different geographical areas with different gene pools [48,49].

Macroblepharon with extreme ectropion resulting in kinked distortion of the central portion of the eyelid, often called “diamond eye” or “pagoda” conformation, has previously been associated with lateral lower eyelid and lateral canthal entropion [2,3]. In the current study, “diamond eye” was recorded in 9.60% of the cases with ectropion, while concurrent entropion and ectropion was recorded only in 2.21% (67/3,029) of the conformational eyelid disorder cases. This low level of dual recording of entropion and ectropion may reflect a tendency of primary care veterinarians to record only the most dominant disorder feature rather than combining the findings into more complex sets of diagnoses affecting the eyelids.

The breed-related odds ratios reported in the current study for conformational eyelid disorders were highly polarised, with odds ratios for many breeds either very high or very low in comparison to crossbreed dogs. The phenotype of the Husky is widely considered to have remained similar to the ancestral natural canine appearance [50] and it is therefore noteworthy that the Husky featured among the breeds with the lowest odds ratio of conformational eyelid disorders in the current study. This supports the proposition for a close connection between conformational eyelid disorders and indeed other conformation-related disorders carrying severe impact on the welfare of the dog with common unnatural phenotypes such as brachycephaly or excessive skin folds selected by humanity during the invention of dog breeds [51]. It is also interesting to note that several breeds that also retain a more natural canine conformation, e.g., Greyhound, Border Collie, Whippet, had significantly lower odds ratio of conformational eyelid disorders than crossbreds. This raises the intriguing question about whether the modern crossbred dog, that is often held up as the ideal and healthy variant, is really so healthy after all. Alternatively, it could be proposed that the modern crossbred dog simply represents a regression to the mean for all the phenotype-related health issues of the wider purebred dog population. This view is supported by several pieces of research that positions disorder risk across multiple disorders in crossbred dogs as being intermediate between the spread of risks for the wider populations of individual breeds [44,52,53].

Concurring with the current results showing concerningly high odds ratios for conformational eyelid disorders in the Shar Pei (OR 107.38) and Chow Chow (OR 60.05), the US ACVO Blue Book 2022 states that the frequency and form of entropion in Shar-Pei and Chow Chow is a particularly severe problem that has been recognised for decades. ACVO also state that selection by breeders for heavy skin folds is a compounding factor encouraging development of entropion. This suggests a strong relationship between the amount and laxity of skin covering the head and face with conformational eyelid disorders [7]. The UK Kennel Club breed standards state for every breed that ‘absolute soundness is essential’ and that ‘breeders and judges should at all times be careful to avoid obvious conditions or exaggerations which would be detrimental in any way to the health, welfare or soundness of this breed’ [54]. The individual breed standards for several of the predisposed breeds include statements that clearly suggest awareness for many decades by the Kennel Club of these breed-related issues, e.g., “function of eyeball or lid in no way disturbed by surrounding skin, folds or hair” (Shar Pei), “free from entropion” (Shar Pei, Chow Chow), “…eyelids should be reasonably tight…excessive haw must be heavily penalised” (Saint Bernard) [54]. It is noteworthy that although breed standards were updated more than three decades ago in some breeds, e.g., Mastiff, to promote improved eyelid conformation, the reported incidence of entropion and ectropion in the Mastiff has still increased during the last decade [3,7]. The current evidence showing very high odds ratios for conformational eyelid disorders in several breeds that are supported by previous reports is very concerning. This suggests that breeders, judges and indeed the wider dog-acquiring public are either unaware of or are not applying the Kennel Club advisory notice on their breed standards and instead are normalising and celebrating extreme conformation as a desirable phenotype in dogs. A logical consequence of this conclusion would mandatory increased training for show judges on assessing those anatomic features that are associated with increased probability for ocular disease in dogs. Severe sanctions could be placed on judges who continue to award dogs with conformational eyelid disorder in the show ring. Additionally, given that the current legislation in England states that *‘…no dog may be kept for breeding if it can reasonably be expected, on the basis of its genotype, phenotype or state of health that breeding from it could have a detrimental effect on its health or welfare or the health or welfare of its offspring*’ [31], then the current results suggest that breeding from dogs with phenotypes such as entropion or ectropion that carry severely detrimental effects on health is in breach of current legislation in several breeds.

The odds ratio for diagnosis with conformational eyelid disorders in the current study reduced greatly with increasing age. This may result from the abnormal eyelid anatomy and conformation being present from birth and therefore generally manifesting with clinical signs that result in diagnosis at early age [2,3]. While under ongoing veterinary care later in life, the diagnosis may then be integrated into the patient’s ‘natural’ problem list and thus no longer perceived as needing recurring acknowledgement as a formal diagnosis in later appointments. While congenital entropion is described to affect dogs from as early as 6 weeks to 2 years of age, a proportion of dogs are also described to “grow out” of mild disorders during maturation to adult conformation [2,3]. This ‘growing out’ has also been described in humans where entropion is described as highly prevalent in Asian children but with a decreasing prevalence by age-group down to one tenth from the percentage in toddlers as the milder cases self-resolve with facial bone growth during the juvenile development phase [55]. However, it could be argued that ‘growing out’ of entropion may be less common in dogs than in humans because trichiasis-associated discomfort in these younger dogs could trigger spastic entropion that leads to further deterioration instead [2]. Ectropion, on the other hand, may potentially worsen with age in dogs: in humans, involuntary ectropion may present as the result of age-related reduction of collagen and elastic fibers disruption and causing further tissue laxity [56,57]. This can potentially explain the more undulating curve in the age at first diagnosis of ectropion compared with entropion in the current study.

The current data show brachycephalic dogs overall with an increased odds ratio for entropion (OR 1.83). This odds ratio was further exaggerated in the subset of those severely brachycephalic breeds. Previously, the probability of corneal ulceration has been strongly linked to the brachycephalic conformation and the existence of a nasal fold [58,59], which are commonly interlinked conformational qualities in severely brachycephalic breeds such as Pekingese, Pug, Shih Tzu and English Bulldog [2,20,58,60,61]. In these breeds, the entropion is common in the medial part of the eyelid [1,2,62], and has been associated with an overly tight medial canthal ligament [63]. A recent study further revealed a possible connection with this phenomenon and a stronger traction rather than length of collagenous attachments between the medial eyelids and lacrimal bone [64]. Co-occurrence of a nasal fold with brachycephaly in many breeds can not only directly cause corneal ulcers by the contact-trichiasis [2,20,58,62,65] but may exaggerate the extent of medial entropion by crowding the already limited space in the medial lower eyelid area in dogs with low craniofacial ratio, as seen with high prevalence of entropion in, e.g., Pug, Dogue de Bordeaux, English Bulldogs and Neapolitan Mastiff in the current data. Interactions between conformational extremes suggest some opportunities to reduce the negative health effects by limiting even some of the extreme conformation even if the wider public are still wedded to others. For example, it could be encouraged to breed away from nasal folds even if a degree of brachycephaly is still desired by a subset of people acquiring dogs. The popularity of brachycephalic breeds continues to grow: in 2019, 18% of the dogs in UK were estimated to be brachycephalic, and 54% of these severely brachycephalic [27]. With such demographic shift, the conditions connected to conformation in these breeds and potential for pain and suffering associated with them cannot be over-emphasised.

The impact of pain associated with these conformational eyelid disorders is a key welfare issue. In two recent studies, brachycephalic breeds were identified with markedly increased odds ratios for corneal ulceration [58,59]. Moreover, corneal disorders have been noted as the second most common type of disorder recorded overall in Pugs [17], while French Bulldogs were stated to have an `ultra-predisposition´ for corneal ulcers [21], and ophthalmological disorders were the second most common group of disorders in English Bulldogs [18]. In the UK, among owners of English Bulldogs, French Bulldogs and Pugs, 8% reported their dog had undergone eyelid surgery [66]. Based on analysis of veterinary clinical records in the UK, pain was recorded for 46% of the dogs diagnosed with corneal ulceration, and an alarming 13% of the deaths reported over the span of the study involved euthanasia decision-making associated with this condition [59]. In the current study, a notable 27% of dogs with entropion and 9% of dogs with ectropion were co-morbidly diagnosed with corneal ulceration. Despite this high level of co-occurrence of such a painful condition, this result may still substantially under-represent the full suffering of dogs with conformational eyelid disorders because it does not include the constant and chronic trichiasis-associated irritation that these dogs endure even in the absence of full-blown corneal ulceration. Unless surgically managed, conformational eyelid disorders are typically chronic in nature and it is reasonable to assume that most of these dogs suffer from chronic pain, which in turn has been associated with changes in behavior, learning and performance [67].

In spaniel breeds, the amount and weight of the skin covering the head and face have been linked with increased probability of conformational eyelid disorders [7]. The current study also identified that increasing bodyweight was associated with increasing odds ratio for ectropion. This finding aligns with the previous reports indicating large- and giant-breed dogs and also breeds with oversized palpebral fissure, including many spaniel breeds, are prone to ectropion and “diamond eye”-conformation [2,3,7]. A small but significant difference in the orientation of the lateral canthal tendon in mesocephalic breeds in comparison to dolichocephalic has been described as resulting in lateral canthal entropion in dogs with redundant facial skin [5]. This is evident for example in Saint Bernard, a breed that often presents with overly long eyelids, “diamond eye”-conformation and ectropion, all of which further result in lateral lower (and at times also upper) eyelid entropion. Concurrent lateral canthal laxity and entropion further complicates this combination of disorders [2,3]. In the current study, those breeds with mesocephalic skull conformation, erect or semi-erect ears and no excess skin or folding that most resemble the ancestral natural canine appearance, e.g., Husky, Border Collie and many terrier breeds, were typical of the most breeds that were most protected from conformational eyelid disorders.

Interestingly, several of those breeds identified with high odds ratios for ectropion reported in the current study such as Neapolitan Mastiff, Saint Bernard, Cane Corso and English Bulldog have also been reported with a high odds ratios for prolapsed nictitating membrane gland (PNMG) [25]. As previously discussed, these breeds are the ones often presented with overly long eyelids, suboptimal lateral canthal support and even the “diamond eye”. Over 30 years ago, Saint Bernards were reported with predisposition to hypertrophy of the nictitating membrane gland secondary to the conformational ectropion, a condition speculated to potentially predispose to PNMG [3,68,69]. Fast forward three decades to 2020 and 25% of the dogs undergoing surgery for PNMG are reported to have a concurrent conformational eyelid disorder, while 70% of those were giant breed dogs [70]. PNMG is a condition potentially resulting in chronic inflammation, quantitative dry eye and other secondary, potentially painful complications if left untreated, and thus requires surgical intervention [71,72]. Therefore, the current findings add further evidence of the welfare-threatening effects of conformational eyelid disorders and also suggest that little is changing on overall breed risk over time and that little will change until the normalisation of extreme eyelid conformation is removed.

### Limitations

This paper had several limitations. Some of the breeds listed in the current study are newly invented breeds that are not yet formally recognised by most kennel clubs, but belong to the increasingly popular group of purposely bred “designer” crossbreeds [27]. Designer breeds are really a broad concept, with each specific designer breed resulting from cross-breeding between two or more from a long list of conventional purebreds. Consequently, designer breeds are likely to comprise a wide variety of phenotypes and thus it is not appropriate to draw firm conclusions on overall designer health from the current health. However, it is noteworthy that three of the four designer breeds in the current study (Cockapoo, Cavapoo and Lurcher) showed reduced odds ratios compared to crossbred dogs while the fourth (Labradoodle) was not significantly different in odds ratio. As novel breeds, there is still very limited evidence on the health status for each individual designer breed [53,73] but the current results do provide some evidence of protection to conformation-related disorder in designer crossbred breeds. This may reflect that eyelid conformational disorders represent an extreme conformation that has been deliberately selected for in many breeds [26]. It is possible therefore that designer crossbreed dogs benefit from regression to the mean between the conformations of their progenitor breeds and therefore generally suffer less exaggerated conformation than a progenitor breed with an extreme conformation.

The current study applied secondary use as a research resource to primary care clinical data that were not originally recorded for research purposes. While giving access to large volumes of clinical data on the wider population of dogs under primary veterinary care in the UK, these data were subject to varying levels of clinical acumen and experience and to differing data recording habits among the veterinarians within the study [16]. Specifically in relation to the current work, the study assumed consistent recording of conformational eyelid disorders in dogs by the attending veterinary surgeons. It is possible that perceptions of normality of entropion and ectropion by some veterinarians in breeds where these disorders are still currently considered as desirable traits may have led to under-reporting and hence to under-estimates of the frequency of these disorders. Although the current study has reported on the frequency of conformational eyelid disorders and a range of comorbid ocular conditions in dogs under primary veterinary care in the UK, the detail available in the clinical records did not permit consistent categorization of each disorder as congenital or not.

## Conclusions

In conclusion, over six decades ago in 1963, S.F.J Hodgman talked about presumed hereditary defects in pedigree dogs and stated that “all the legislation in the world will not alone overcome this problem. There is no royal road to success, it is only attained by knowledge, perseverance, hard work and the courageous acceptance of many disappointments” [74]. The current results highlight that serious welfare issues of conformational eyelid disorders still exist in certain dog breeds and provide further evidence for brachycephalism and excessive skin fold as predisposing factors. Overall, it seems that there is still a long way to travel on the road to ensuring good innate health for all dogs.

## Supporting information

S1 Table Clinical signs recorded for cases of en*tropion* during 2019 in dogs under primary veterinary care in the VetCompass Programme in the UK. N = 2,275. (DOCX)

S2 Table Comorbid ocular conditions recorded for cases of en*tropion* during 2019 in dogs under primary veterinary care in the VetCompass™ Programme in the UK. N = 2,275. (DOCX)

S3 Table Clinical management plans recorded in the clinical records at first diagnosis of en*tropion* during 2019 in dogs under primary veterinary care in the VetCompass™ Programme in vcx (DOCX)

S4 Table Surgical management at first surgical intervention for en*tropion* diagnosed during 2019 in dogs under primary veterinary care in the VetCompass™ Programme in the UK. N = 216. (DOCX)

S5 Table Clinical signs recorded for cases of ec*tropion* during 2019 in dogs under primary veterinary care in the VetCompass™ Programme in the UK. N = 305. (DOCX)

S6 Table Comorbid ocular conditions recorded for cases of ec*tropion* during 2019 in dogs under primary veterinary care in the VetCompass™ Programme in the UK. N = 305. (DOCX)

S7 Table Clinical management plans recorded in the clinical records at first diagnosis of ec*tropion* during 2019 in dogs under primary veterinary care in the VetCompass™ Programme in the UK. N = 305. (DOCX)

## Abbreviations

ACVO: American College of Veterinary Ophthalmologists
CI: confidence interval
ECVO: European College of Veterinary Ophthalmologist
EHR: Electronic health record
IQR: Interquartile range
KC: Kennel Club
OR: odds ratio
PNMG: prolapsed nictitating membrane gland
