# Supplementary material for: Conformational eyelid disorders in dogs under primary veterinary care in the UK - Epidemiology and clinical management
Source: PLoS One. 2025 Jun 30;20(6):e0326526. doi: 10.1371/journal.pone.0326526 (PMC12208470; doi:10.1371/journal.pone.0326526)
Supplement: S1 Table — (DOCX) [file pone.0326526.s001.docx]

Supplementary 1 Table. Clinical signs recorded for cases of en*tropion* during 2019 in dogs under primary veterinary care in the VetCompass Programme in the UK. N = 2,275

| Clinical sign | No. | % [1540] |
| --- | --- | --- |
| None mentioned | 735 |  |
| Epiphora | 428 | 27.79 |
| Squinting/blepharospasm | 353 | 22.92 |
| Discharge, not specified | 265 | 17.21 |
| Discharge, purulent | 192 | 12.47 |
| Discharge, mucus | 187 | 12.14 |
| Conjunctival redness/hyperemia | 153 | 9.94 |
| Swelling | 147 | 9.55 |
| Chemosis | 95 | 6.17 |
| Discharge, serous | 91 | 5.91 |
| Neovascularisation | 89 | 5.78 |
| Corneal pigmentation | 82 | 5.32 |
| Corneal edema | 77 | 5.00 |
| Corneal opacities | 67 | 4.35 |
| Corneal fibrosis | 57 | 3.70 |
| Unspecified eye inflammation | 42 | 2.73 |
| Periocular inflammation | 38 | 2.47 |
| Alopecia around eye | 37 | 2.40 |
| Pigmentary keratitis | 37 | 2.40 |
| Prolapsed nictitating membrane gland | 29 | 1.88 |
| Protruding third eyelid | 29 | 1.88 |
| Enophthalmia | 21 | 1.36 |
| Erythema around eye | 19 | 1.23 |
| Granulation tissue formation | 18 | 1.17 |
| Scleral congestion | 15 | 0.97 |
| Scleral hyperemia | 11 | 0.71 |
| Periocular lichenification | 10 | 0.65 |
| Episcleral congestion | 9 | 0.58 |
| Conjunctival congestion | 8 | 0.52 |
| Episcleral injection | 8 | 0.52 |
| Face muscle atrophy | 8 | 0.52 |
| Exophthalmos | 7 | 0.45 |
| Scleral pigmentation | 4 | 0.26 |
| Pigmentation at medial canthus | 3 | 0.19 |
| Horner's syndrome | 2 | 0.13 |
| Scleral edema | 2 | 0.13 |
| Scleral pigmentation | 2 | 0.13 |
| Anisocoria | 1 | 0.06 |
| Buphthalmia | 1 | 0.06 |
| Conjunctival injection | 1 | 0.06 |
| Episcleral pigmentation | 1 | 0.06 |
| Facial edema | 1 | 0.06 |
| Hyperkeratosis of eyelid | 1 | 0.06 |
| Hypopyon | 1 | 0.06 |
| Ptosis | 1 | 0.06 |
| Reduced IOP | 1 | 0.06 |
| Scabbing of eyelid | 1 | 0.06 |
| Aqueous flare | 0 | 0.00 |
| Corneal lipidosis | 0 | 0.00 |
