# Supplementary material for: Conformational eyelid disorders in dogs under primary veterinary care in the UK - Epidemiology and clinical management
Source: PLoS One. 2025 Jun 30;20(6):e0326526. doi: 10.1371/journal.pone.0326526 (PMC12208470; doi:10.1371/journal.pone.0326526)
Supplement: S2 Table — (DOCX) [file pone.0326526.s002.docx]

Supplementary 2 Table. Comorbid ocular conditions recorded for cases of en*tropion* during 2019 in dogs under primary veterinary care in the VetCompass™ Programme in the UK. N = 2,275

| Entropion - Comorbid condition | No. | % [1019] |
| --- | --- | --- |
| Conjunctivitis | 414 | 40.63 |
| Corneal ulceration | 278 | 27.28 |
| Trichiasis | 104 | 10.21 |
| Not possible to assess due to behavior | 96 | 9.42 |
| Distichiasis | 63 | 6.18 |
| Blepharitis | 46 | 4.51 |
| Diamond eye | 40 | 3.93 |
| Keratitis | 40 | 3.93 |
| Dry eye, quantitative=KCS | 35 | 3.43 |
| Dry eye, unspecified | 25 | 2.45 |
| Mass on eyelid | 24 | 2.36 |
| Microphthalmia | 17 | 1.67 |
| Macropalpebral fissure | 17 | 1.67 |
| Lagophthalmos | 15 | 1.47 |
| Ectopic cilia | 15 | 1.47 |
| Scleritis | 15 | 1.47 |
| Macroblepharon | 14 | 1.37 |
| Dacryostenosis | 13 | 1.28 |
| Excessive eyelid length | 11 | 1.08 |
| Uveitis | 9 | 0.88 |
| Skin fold in contact with cornea | 7 | 0.69 |
| Episcleritis | 5 | 0.49 |
| Dry eye, qualitative | 4 | 0.39 |
| Micropalpebral fissure | 4 | 0.39 |
| Corneal perforation | 3 | 0.29 |
| Trauma to eyelid | 3 | 0.29 |
| Euryblepharon | 2 | 0.20 |
| Asteroid hyalosis | 1 | 0.10 |
| Hairy caruncle | 1 | 0.10 |
| Foreign body behind 3rd eyelid | 1 | 0.10 |
| Pyoderma at medial canthus | 1 | 0.10 |
| Blocked meibomian gland | 1 | 0.10 |
| Cartilage eversion in eye | 1 | 0.10 |
| Exposure keratopathy | 1 | 0.10 |
| Microcornea | 1 | 0.10 |
| Microblepharon | 1 | 0.10 |
| Keratomalacia | 0 | 0.00 |
| Descemetocele | 0 | 0.00 |
| 3rd eyelid trauma | 0 | 0.00 |
| None mentioned | 1256 |  |
