# Supplementary material for: Conformational eyelid disorders in dogs under primary veterinary care in the UK - Epidemiology and clinical management
Source: PLoS One. 2025 Jun 30;20(6):e0326526. doi: 10.1371/journal.pone.0326526 (PMC12208470; doi:10.1371/journal.pone.0326526)
Supplement: S3 Table — (DOCX) [file pone.0326526.s003.docx]

Supplementary 3 Table. Clinical management plans recorded in the clinical records at first diagnosis of en*tropion* during 2019 in dogs under primary veterinary care in the VetCompass™ Programme in the UK. N = 2,275

| ENTROPION: Management plan on the day of earliest diagnosis | No. | % [1906] |
| --- | --- | --- |
| Medical | 1133 | 59.44 |
| Discuss surgery | 1098 | 57.61 |
| Wait and see | 266 | 13.96 |
| Referral | 180 | 9.44 |
| Assess under sedation | 39 | 2.05 |
| Refer in house to another vet | 13 | 0.68 |
| Euthanasia | 3 | 0.16 |
| Plan not discussed in clinical notes | 369 |  |
