# Supplementary material for: Conformational eyelid disorders in dogs under primary veterinary care in the UK - Epidemiology and clinical management
Source: PLoS One. 2025 Jun 30;20(6):e0326526. doi: 10.1371/journal.pone.0326526 (PMC12208470; doi:10.1371/journal.pone.0326526)
Supplement: S4 Table — (DOCX) [file pone.0326526.s004.docx]

Supplementary 4 Table. Surgical management at first surgical intervention for en*tropion* diagnosed during 2019 in dogs under primary veterinary care in the VetCompass™ Programme in the UK. N = 216

| ENTROPION: If surgical treatment, what technique was used for the first surgery | No. | % [216] |
| --- | --- | --- |
| Hotz-Celsus | 86 | 39.81 |
| Stay sutures | 83 | 38.43 |
| Wedge resection | 28 | 12.96 |
| Modified Celsus-Hotz | 17 | 7.87 |
| Medial canthoplasty | 13 | 6.02 |
| Arrowhead | 11 | 5.09 |
| Stades procedure | 6 | 2.78 |
| Lateral canthoplasty | 4 | 1.85 |
| Tarsorraphy | 2 | 0.93 |
| Enucleation | 2 | 0.93 |
| Bigelbach lateral canthoplasty | 1 | 0.46 |
| Munger and Carter's | 1 | 0.46 |
| Modified Khunt-Szymanowski | 1 | 0.462 |
| Other | 0 | 0 |
| Unspecified | 198 |  |
