# Supplementary material for: Conformational eyelid disorders in dogs under primary veterinary care in the UK - Epidemiology and clinical management
Source: PLoS One. 2025 Jun 30;20(6):e0326526. doi: 10.1371/journal.pone.0326526 (PMC12208470; doi:10.1371/journal.pone.0326526)
Supplement: S5 Table — (DOCX) [file pone.0326526.s005.docx]

Supplementary 5 Table. Clinical signs recorded for cases of ec*tropion* during 2019 in dogs under primary veterinary care in the VetCompass™ Programme in the UK. N = 305

| ECTROPION: Clinical signs recorded on the day of earliest diagnosis for incident cases in 2019 | No. | % [167] |
| --- | --- | --- |
| Conjunctival redness/hyperemia | 46 | 27.54 |
| Discharge | 34 | 20.36 |
| Discharge, not specified | 33 | 19.76 |
| Discharge, purulent | 25 | 14.97 |
| Epiphora | 25 | 14.97 |
| Protruding third eyelid | 14 | 8.38 |
| Chemosis | 10 | 5.99 |
| Corneal opacities | 10 | 5.99 |
| Discharge, serous | 9 | 5.39 |
| Swelling | 9 | 5.39 |
| Neovascularisation | 7 | 4.19 |
| Prolapsed nictitating membrane gland | 7 | 4.19 |
| Corneal fibrosis | 6 | 3.59 |
| Enophthalmia | 6 | 3.59 |
| Squinting/blepharospasm | 6 | 3.59 |
| Alopecia around eye | 2 | 1.20 |
| Conjunctival congestion | 2 | 1.20 |
| Corneal edema | 2 | 1.20 |
| Corneal pigmentation | 2 | 1.20 |
| Granulation tissue formation | 2 | 1.20 |
| Periocular lichenification | 2 | 1.20 |
| Ptosis | 2 | 1.20 |
| Scleral injection | 2 | 1.20 |
| Episcleral redness/hyperemia | 1 | 0.60 |
| Erythema around eye | 1 | 0.60 |
| Periocular inflammation | 1 | 0.60 |
| Temporal muscle atrophy | 1 | 0.60 |
| Unspecified eye inflammation | 1 | 0.60 |
| Aqueous flare | 0 | 0.00 |
| Dry skin around eye | 0 | 0.00 |
| Globe retraction | 0 | 0.00 |
| Hyphema | 0 | 0.00 |
| Hypopyon | 0 | 0.00 |
| Pigmentary keratitis | 0 | 0.00 |
| None mentioned | 138 |  |
