# Supplementary material for: Conformational eyelid disorders in dogs under primary veterinary care in the UK - Epidemiology and clinical management
Source: PLoS One. 2025 Jun 30;20(6):e0326526. doi: 10.1371/journal.pone.0326526 (PMC12208470; doi:10.1371/journal.pone.0326526)
Supplement: S6 Table — (DOCX) [file pone.0326526.s006.docx]

Supplementary 6 Table. Comorbid ocular conditions recorded for cases of ec*tropion* during 2019 in dogs under primary veterinary care in the VetCompass™ Programme in the UK. N = 305

| ECTROPION: Comorbid conditions for incident conditions in 2019 | No. | % [177] |
| --- | --- | --- |
| Conjunctivitis | 55 | 31.07 |
| Corneal ulceration | 16 | 9.04 |
| Mass on eyelid | 11 | 6.21 |
| Not possible to assess due to behavior | 10 | 5.65 |
| Dry eye, quantitative = KCS | 8 | 4.52 |
| Distichiasis | 7 | 3.95 |
| Excessive eyelid length | 4 | 2.26 |
| Dry eye, unspecified | 2 | 1.13 |
| Horner's syndrome | 2 | 1.13 |
| Keratitis | 2 | 1.13 |
| Microphthalmia | 2 | 1.13 |
| Trauma on eyelid | 2 | 1.13 |
| Blepharitis | 1 | 0.56 |
| Dacryostenosis | 1 | 0.56 |
| Euryblepharon | 1 | 0.56 |
| Macroblepharon | 1 | 0.56 |
| Macropalpebral fissure | 1 | 0.56 |
| Mass on eye (excluding dermoid) | 1 | 0.56 |
| Trichiasis | 1 | 0.56 |
| Descemetocele | 0 | 0.00 |
| Dry eye, qualitative | 0 | 0.00 |
| Ectopic cilia | 0 | 0.00 |
| Keratomalacia | 0 | 0.00 |
| Lagophthalmos | 0 | 0.00 |
| Skin fold in contact with cornea | 0 | 0.00 |
| None mentioned | 188 |  |
