# Supplementary material for: Conformational eyelid disorders in dogs under primary veterinary care in the UK - Epidemiology and clinical management
Source: PLoS One. 2025 Jun 30;20(6):e0326526. doi: 10.1371/journal.pone.0326526 (PMC12208470; doi:10.1371/journal.pone.0326526)
Supplement: S7 Table — (DOCX) [file pone.0326526.s007.docx]

Supplementary 7 Table. Clinical management plans recorded in the clinical records at first diagnosis of ec*tropion* during 2019 in dogs under primary veterinary care in the VetCompass™ Programme in the UK. N = 305

| ECTROPION: Management plan on the day of earliest diagnosis | No. | % [201] |
| --- | --- | --- |
| Medical | 121 | 60.20 |
| Discuss surgery | 65 | 32.34 |
| Wait and see | 58 | 28.86 |
| Referral | 14 | 6.97 |
| Assess under sedation | 1 | 0.50 |
| Refer internally to another vet | 1 | 0.50 |
| Plan not discussed in clinical notes | 104 |  |
